# Prenatal Diagnosis of Autosomal Recessive Primary Microcephaly Type 2 Caused by Compound Heterozygous *WDR62* Variants in a Family With Two Recurrent Cases

**DOI:** 10.1002/mgg3.70203

**Published:** 2026-04-19

**Authors:** Yan‐Fang Li, Song‐Hui Zhang, Li Zhen, Lan‐Zhen Zhang

**Affiliations:** ^1^ Department of Obstetrics The Second Affiliated Hospital of Guangzhou Medical University Guangzhou Guangdong Province China; ^2^ Prenatal Diagnostic Center Guangdong Second Provincial General Hospital Guangzhou Guangdong Province China

**Keywords:** cortical malformations, MCPH2, microcephaly, *WDR62* gene, whole‐exome sequencing

## Abstract

**Objective:**

Autosomal recessive microcephaly type 2 (MCPH2), caused by biallelic *WDR62* variants, is a rare neurodevelopmental disorder typically described postnatally. We aimed to delineate its prenatal phenotype via data from two affected fetuses in a family.

**Methods:**

Trio whole‐exome sequencing (WES) was performed on one fetus and his parents. Variants were prioritized via population databases, computational predictions, and segregation analysis, which were confirmed via Sanger sequencing.

**Results:**

Prenatal imaging revealed microcephaly, agenesis of the corpus callosum, and neuronal migration defects (lobar holoprosencephaly and lissencephaly) in two fetuses. Trio‐WES identified compound heterozygous *WDR62* variants (c.1128C > A/p.Cys376* and c.643A > C/p.Thr215Pro) in one fetus, each inherited from a heterozygous parent. Sanger sequencing confirmed variants in both fetuses and parents.

**Conclusions:**

Trio‐WES is crucial for the prenatal diagnosis of fetuses with unresolved sonographic anomalies. We report the prenatal features and molecular diagnoses of MCPH2, expanding its prenatal phenotypic spectrum and enhancing its clinical genomic database utility.

## Graphical abstract

We report prenatal diagnosis of MCPH2 via trio‐WES, identifying novel compound heterozygous *WDR62* variants. To our knowledge, prenatal MRI detection of lobar HPE and lissencephaly with the agyria–pachygyria complex has not been reported in fetuses with *WDR62* variants. Consequently, these findings have advanced our understanding of prenatally detectable phenotypes linked to *WDR62* variants.

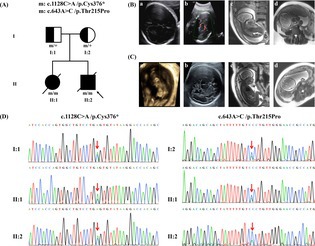

## 1 Background

Autosomal recessive primary microcephaly 2 (MCPH2; MIM:604317) exhibits significant epidemiological heterogeneity, with a global prevalence ranging from 1.3–150 per 100,000 live births (Naseer et al. 2017). This disparity predominantly reflects variations in population genetic architecture and regional consanguinity. The clinical diagnosis of MCPH2 requires documentation of head circumference (HC) falling 3 standard deviations (SD) below the age‐ and sex‐adjusted mean, with or without cortical malformations, and variable severity of intellectual disability (Shaheen et al. 2019; Cherkaoui Jaouad et al. 2018).

The established genetic cause of MCPH2 is a variant in the *WDR62* gene (WD repeat‐containing protein 62; MIM:613583). First identified by Bilguvar et al. in 2010 (Bilgüvar et al. 2010), the gene is located on chromosome 19q13.12, spans 50,230 bp, and comprises 32 coding exons. It encodes a full‐length protein of 1523 amino acid residues containing multiple WD40 repeat (WDR) domains at the *N*‐terminus (Roberts et al. 1999; Kousar et al. 2011). Present in diverse eukaryotic proteins, WDR domains mediate key cellular functions such as signal transduction, pre‐mRNA processing, and cytoskeletal assembly regulation, primarily by serving as rigid scaffolds for multiprotein complex formation. The gene is expressed in neural progenitor cells and postmitotic neurons within the developing brain, particularly in the ventricular and subventricular zones of the forebrain. *WDR62* encodes a centrosome‐associated protein crucial for orchestrating neurogenesis and cerebral cortical morphogenesis (Nicholas et al. 2010). Its functions include regulating neuronal proliferation and migration (Yu et al. 2010), participating in the c‐Jun N‐terminal kinase 2 (JNK2) pathway (Wasserman et al. 2010), and interacting genetically with Aurora A kinase to control spindle formation, mitotic progression, and ultimately, brain size (Sgourdou et al. 2017; Carmena et al. 2009). Disruption of *WDR62* or its interactomes, such as with Aurora kinases, leads to mitotic delay and death of neural progenitor cells (NPCs), representing a key pathogenic mechanism underlying human microcephaly (Chen et al. 2014).

Individuals harboring *WDR62* pathogenic variants exhibit a broad spectrum of dysmorphic features (Table S1) with considerable interindividual variability. Common features include a sloping forehead, micrognathia, low‐set and/or posteriorly rotated ears, a high‐arched palate, abnormal dermatoglyphics, and brachydactyly (Bhat et al. 2011). Affected individuals also display diverse neurological and behavioral manifestations, ranging from reduced fetal movements and delayed psychomotor development to intellectual disability, behavioral disturbances (e.g., impulsivity, aggression), and epilepsy (Ruaud et al. 2022). In addition to microcephaly, associated brain malformations include callosal abnormalities, polymicrogyria, schizencephaly, and subcortical nodular heterotopia (Bilgüvar et al. 2010). However, detailed prenatal characterization of fetal anomalies beyond microcephaly, particularly via ultrasound and magnetic resonance imaging (MRI), remains markedly limited in reported MCPH2 cases.

In this study, we report a family with recurrent fetal structural anomalies identified prenatally via both ultrasound and magnetic resonance imaging (MRI). These anomalies are characterized by microcephaly, agenesis of the corpus callosum (ACC), and neuronal migration defects manifested as lobar holoprosencephaly (HPE) and lissencephaly (agyria–pachygyria complex). Through WES, we identified compound heterozygous variants in the *WDR62* gene: c.1128C > A (p.Cys376*) and c.643A > C (p.Thr215Pro). To our knowledge, prenatal MRI detection of lobar HPE and lissencephaly with the agyria–pachygyria complex has not been reported in fetuses with *WDR62* variants. Consequently, these findings have advanced our understanding of prenatally detectable phenotypes linked to *WDR62* variants.

## 2 Materials and Methods

### 2.1 Clinical Report

This study was approved by the Institutional Review Board of the Second Affiliated Hospital of Guangzhou Medical University. Written informed consent was obtained from legal guardians for publication of any potentially identifiable images or data included in this research.

**FIGURE 1:**
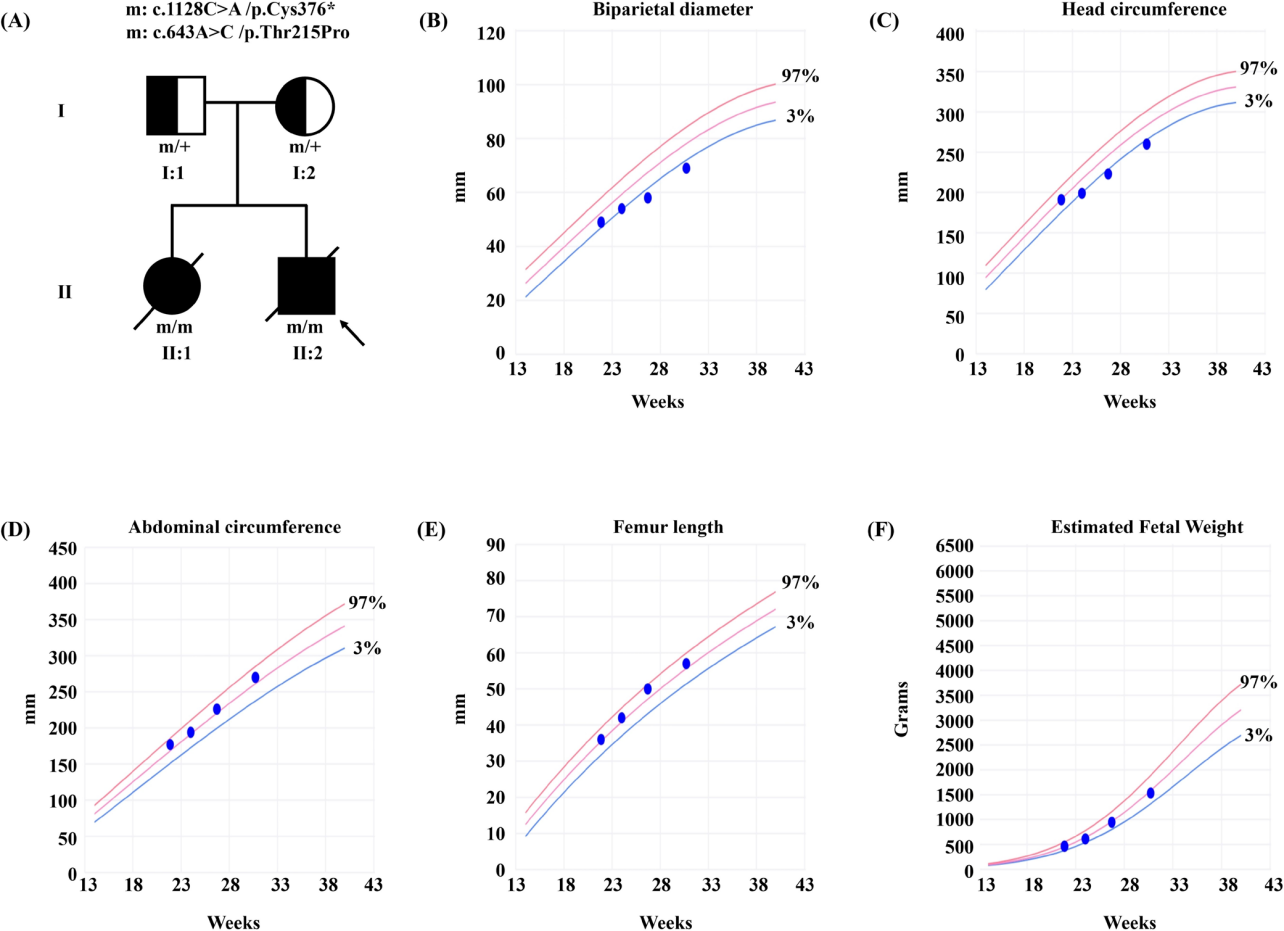
Family pedigree and fetal growth parameters assessed by ultrasound. (A) Pedigree of the family. Heterozygous parents are denoted asm/+ the fetus with compound heterozygous variants is indicated as m/m. The proband is marked by a black arrow. (B–F) Serial biometric measurements, with growth curves referenced to a Chinese population standard, demonstrating progressive fetal growth restriction.

**FIGURE 2:**
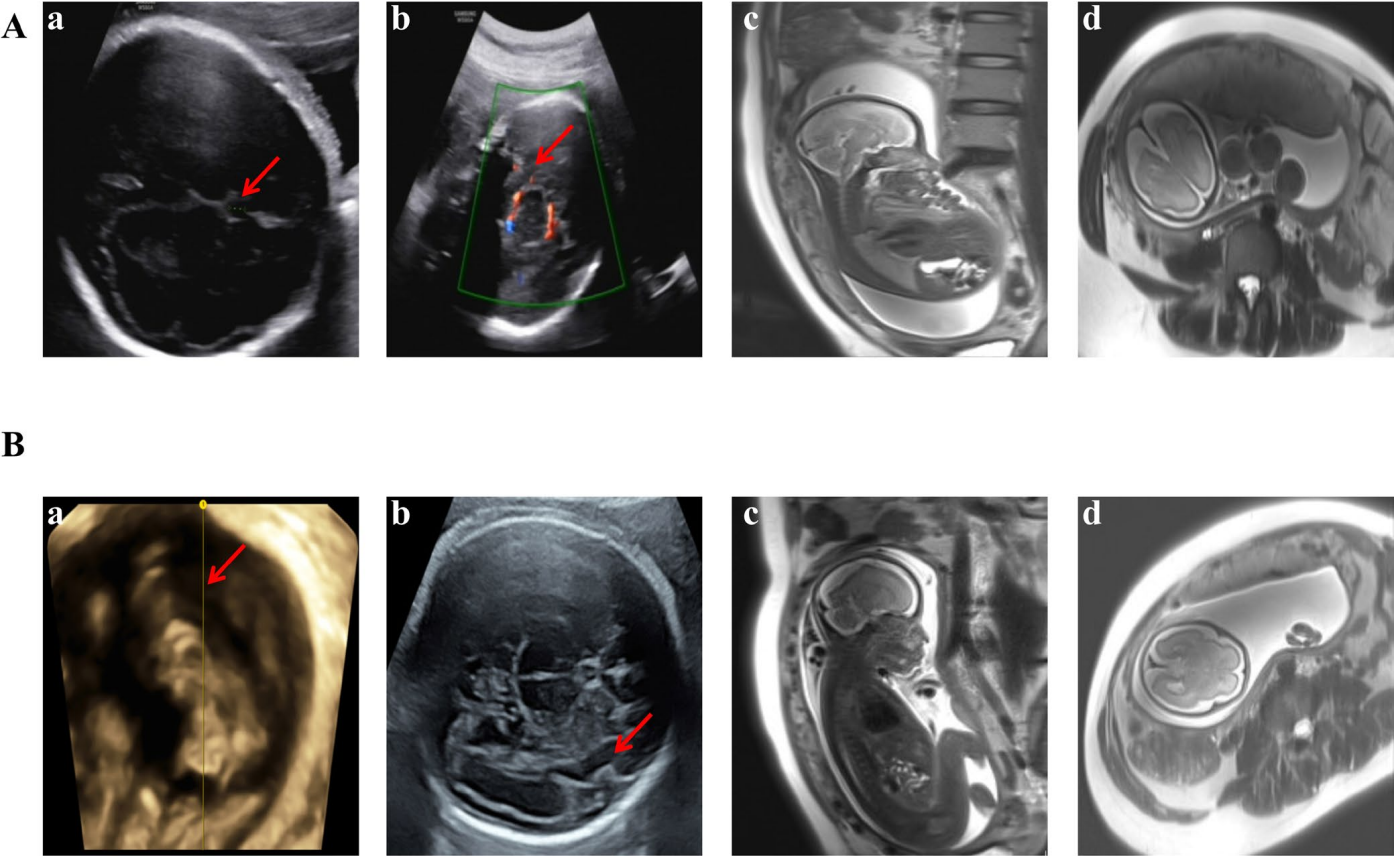
Prenatal ultrasound and MRI findings (A) Fetus II:1. (a, b) Ultrasound at 29^+6^ weeks’ gestation (WG) showed a reduced cavum septi pellucidi (CSP) and agenesis of the corpus callosum (ACC). (c, d) MRI at 30 WG confirmed absent septum pellucidum and corpus callosum, with poor visualization of the anterior horns of the lateral ventricles and optic chiasm. Additional findings included mild hypoplasia of the parieto‐occipital lobes, mild frontal cortical thickening, and an indistinct frontothalamic boundary, suggestive of lobar holoprosencephaly (HPE). (B) Fetus II:2. (a) Ultrasound at 26^+6^ WG indicated partial ACC. (b) Ultrasound at 30^+5^ WG revealed cortical dysplasia with closed Sylvian fissures, simplified gyration, and shallow sulci. (c, d) MRI at 28 WG demonstrated microcephaly, partial ACC, and bilateral cerebral hemispheric abnormalities consistent with a neuronal migration disorder (agyria–pachygyria complex).

A 35‐year‐old gravida 2 para 0 Chinese Han woman (Figure 1A; I:2) with no significant medical history presented for prenatal care. She denied any history of substance misuse or travel to Zika‐endemic regions. Both the patient and her nonconsanguineous husband (Figure 1A; I:1) presented normal cephalic parameters and neurodevelopment. Her first pregnancy (Figure 1A; II:1), prenatal ultrasound at 29^+6^ WG revealed fetal microcephaly and diminished cavum septi pellucidi (CSP) and ACC (Figure 2A; a–b). Fetal MRI at 30 WG confirmed severe microcephaly, complete ACC, and lobar HPE (Figure 2A; c‐d). Maternal serological testing was negative for Toxoplasma immunoglobulin M, and both maternal urine and amniotic fluid cytomegalovirus tests were negative. Chromosomal microarray (CMA) of amniotic fluid revealed no pathogenic copy number variants. Following parental declination of Trio‐WES data, the pregnancy was terminated at 36^+2^ WG. Postmortem examination of the female fetus (2000 g, 45 cm) confirmed complete ACC, absence of the septum pellucidum, and fusion of the anterior horns of the lateral ventricles, which was consistent with prenatal imaging.

In her current (second) pregnancy (Figure 1A; II:2), first‐trimester evaluations were normal, including a 12 WG ultrasound (crown‐rump length 55 mm, nuchal translucency 1.5 mm) and low‐risk noninvasive prenatal testing (NIPT) at 13 WG. Progressive microcephaly (Table 1; Figure 1B–F) developed in the second trimester: HC measured 191 mm at 21^+6^ WG and fell below the 2.5th percentile (−2.0 SD) at 199 mm by 24 WG. Specialist ultrasound at 26^+6^ WG confirmed microcephaly (biparietal diameter [BPD] < 0.3rd percentile [−3.0 SD]; HC < 0.6th percentile [−2.5 SD]) and partial ACC (Figure 2B; a). Amniocentesis at 27^+1^ WG revealed a normal karyotype and CMA, and all IgM serological tests for recent fetal infections were negative. Fetal MRI (Figure 2B; c–d) at 28 WG revealed microcephaly, partial ACC, and lissencephaly (agyria‐pachygyria complex). Third‐trimester ultrasound at 30^+5^ WG confirmed persistent severe microcephaly (BPD < 0.3rd percentile [−2.8 SD]; HC < 0.7th percentile [−2.5 SD]) with cortical dysplasia, characterized by closed Sylvian fissures, simplified gyration, and shallow sulci (Figure 2B; b). Trio‐WES was initiated due to recurrent fetal cortical malformations and negative cytogenetic studies. Following extensive genetic counseling, the pregnancy was terminated at an external facility, precluding postmortem phenotyping.

**TABLE 1:** Intrauterine Parameters of Fetus II:2.

	BPD (mm)	Percentile (SD)	HC (mm)	Percentile (SD)	AC (mm)	Percentile (SD)	FL (mm)	Percentile (SD)	EFW (*g*)	Percentile (SD)
21^+6^	49	11.9% (−1.2)	191	47.2% (−0.1)	177	82.6% (0.9)	36	60.3% (0.3)	464	62.6% (0.3)
24	54	4.0% (−1.7)	199	2.5% (−2)	194	59.1% (0.2)	42	70.9% (0.6)	612	30.9% (−0.5)
26^+5^	58	under0.1% (−3)	223	0.6% (−2.5)	226	68.4% (−0.5)	50	90.3% (1.3)	946	43.6% (−0.2)
30^+5^	69	0.3% (−2.8)	260	0.7% (−2.5)	270	75.5% (−0.7)	57	72.2% (0.6)	1533	37.4% (−0.3)

Abbreviations: AC, abdominal circumference; BPD, biparietal diameter; EFW, estimated fetal weight; FL, femur length; HC, head circumference.

### 2.2 Whole Exome Sequencing

Genomic DNA was extracted from fetal amniotic fluid and parental peripheral blood samples via the QIAamp DNA Blood Mini Kit (QIAGEN, Germany) per the manufacturer's protocol. Trio‐WES was performed on fetal‐parental DNA via the Agilent SureSelect Human Exome Capture Kit (v6; Life Technologies, Waltham, MA, USA) for exonic enrichment. Libraries were sequenced on Illumina platforms (HiSeq X Ten or NovaSeq 6000), generating 150‐bp paired‐end reads, achieving > 99% coverage at 20 × depth (Fu et al. 2018). The raw sequencing data were aligned to the Genome Reference Consortium Human Genome Build 37 (GRCh37) via the Burrows–Wheeler Aligner (BWA) algorithm. Single‐nucleotide variants (SNVs) and insertion/deletion variants (indels) were subsequently identified and functionally annotated via the Genome Analysis Toolkit (GATK). All identified variants were classified as pathogenic (P), likely pathogenic (LP), benign (B), likely benign (LB), or variant of uncertain significance (VUS) in strict accordance with the American College of Medical Genetics and Genomics (ACMG) variant interpretation guidelines (Richards et al. 2015).

### 2.3 Molecular Effects of Candidate Variants

The pathogenicity of candidate missense variants was assessed via web‐based in silico prediction tools (Li et al. 2018). Protein structure visualization, analysis, and rendering were performed via PyMOL (v2.3.2; Schrödinger) (Schiffrin et al. 2020). Structural and functional impacts were evaluated via protein modeling via the iterative thresholding software ASSEMBLES Refinement (I‐TASSER) (Yang and Zhang 2015). The IStable platform was used to predict protein stability changes induced by missense variants (Chen et al. 2013), which were quantified by Gibbs free energy variation (ΔΔG; kcal/mol). ΔΔG values > +0.5 kcal/mol indicated significant structural stabilization, whereas values < −0.5 kcal/mol indicated significant destabilization. Amino acid conservation was analyzed across species via T‐Coffee (Notredame et al. 2000), including *Homo sapiens* (UniProt Entry: A0A7P0TBE7), *Bos taurus* (A0AAA9TMJ), *Rattus norvegicus* (F1M5K2), *Mus musculus* (E9QK36), *Drosophila melanogaster* (M9NE65), *Sus scrofa* (Q8HXL3), *Oryctolagus cuniculus* (G1TFY4), *Ailuropoda melanoleuca* (G1L2A6), *Aotus nancymaae* (A0A2K5F9J6), and *Panthera leo* (A0A8C8XFI0).

### 2.4 Sanger Sequencing Verification of the *WDR62* Gene

Sanger sequencing was conducted to confirm the potential causative variants from this family, which included two fetuses (II:1 and II:2), a father, and a mother. The primers used were designed with Primer 5.0. The primers used were as follows: *WDR62‐*c.1128C > A, forward, 5′‐AGCTTCCTCTTCCACAGG‐3′, reverse, 5′‐CACCTCCACGTTCCAAACG‐3′ and *WDR62*‐c.643A > C, forward, 5′‐AGAAAGACATCGTAGTGGCC‐3′, reverse, 5′‐TCACCTTTGTCTCAGTGG‐3′. The sequence data were analyzed via Sequencing Analysis Software 6 (Applied Biosystems, Foster City, CA, USA).

### 2.5 Genotype–Phenotype Correlation Analysis

To investigate the genotype–phenotype relationship, we performed a systematic review and analysis of *WDR62* gene variants associated with MCPH2 reported in Chinese and English publications. Data were collected through comprehensive searches of the Human Gene Mutation Database (http://www.hgmd.cf.ac.uk/ac/index.php) and the PubMed database (https://pubmed.ncbi.nlm.nih.gov/) up to May 2025. We systematically characterized the types of *WDR62* variants, their localization within the WD40 domain, and comprehensive clinical data from affected patients. Clinical information included geographic origin, sex, prenatal detection of microcephaly, occipitofrontal circumference (OFC) at birth, pregnancy complications, age and OFC at last examination, epilepsy, developmental delay (DD), intellectual disability/mental retardation (ID/MR), dysmorphic features, and radiographic imaging findings.

To assess the association between radiographic imaging findings and two genetic classification schemes, we applied Fisher's exact test. The first scheme categorizes variants by type into four groups: A (one null and one missense variant), B (two missense variants), C (two null variants), and D (other combinations). The second scheme classifies variants by location relative to the WD40 domain into three groups: A (both inside), B (one inside, one outside), and C (both outside). A Bonferroni correction was used to adjust for multiple comparisons.

## 3 Results

### 3.1 Identification of *WDR62* Variants

**FIGURE 3:**
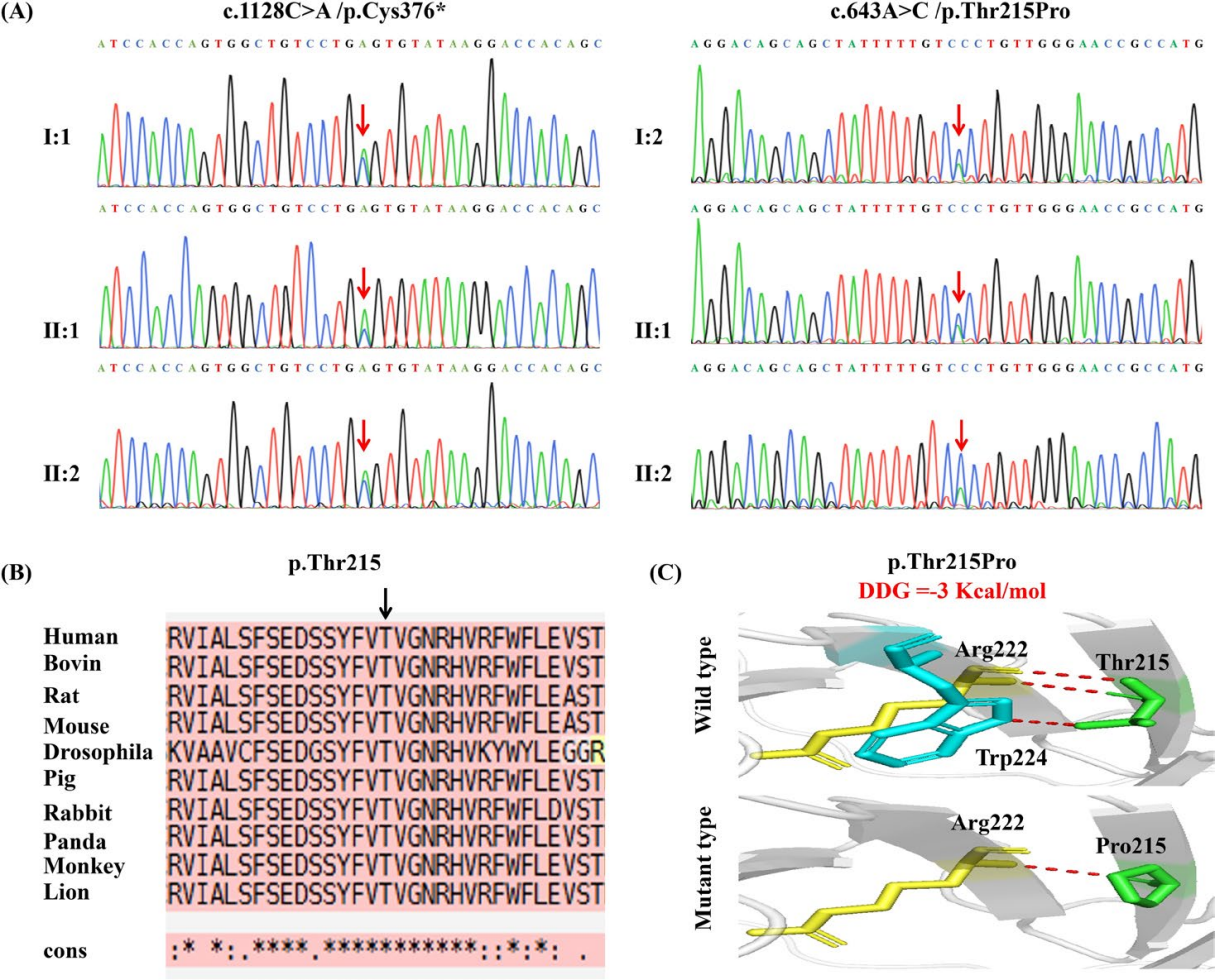
Sanger sequencing validation of the *WDR62* variants and bioinformatics analysis. (A) Sanger sequencing confirmed compound heterozygous *WDR62* variants: Paternal c.1128C > A (p.Cys376Ter) and maternal c.643A > C (p.Thr215Pro) in affected fetuses II:1 and II:2. The variants are indicated by red arrows. (B) Amino acid alignment of the wild‐type protein encoded by the *WDR62* gene. The black arrow shows that amino acid 215 is conserved across these species. (C) Hydrogen bond changes and DDG values of the missense (p.Thr215Pro) variant. Wild‐type Thr215 forms two bonds with Arg222 and one with Trp224, whereas Thr215Pro destroys one hydrogen bond with residues Arg222 and Trp224, respectively.

Trio‐WES was performed on the second fetus (II:2) and both parents (I:1; I:2). Subsequent analysis confirmed that there was no consanguinity between the parents. Sequencing variants were prioritized on the basis of allele frequency, segregation analysis, and in silico algorithmic analysis. Notably, the compound heterozygous variants c.1128C > A (p.Cys376*) and c.643A > C (p.Thr215Pro) in the *WDR62* gene (NM_001083961.2) were identified (Figure 3A; Figure 5). Subsequent Sanger sequencing analysis of the archived DNA sample from the first pregnancy (II:1) revealed the same *WDR62* compound heterozygous variants as expected.

The c.1128C > A variant has an ultrarare allele frequency (MAF < 0.0004%) in the gnomAD database. Bioinformatic analysis predicted that this variant creates a nonsense variant (p.Cys376*) in exon 9 of the *WDR62* gene, introducing a premature termination codon that is likely to trigger nonsense‐mediated mRNA decay through canonical surveillance mechanisms. Sanger sequencing revealed that this variant was inherited from the father (Figure 3A). Accordingly, c.1128C > A (p.Cys376*) was classified as “LP” on the basis of ACMG/AMP criteria: PVS1 and PM2_Supporting.

The c.643A > C variant in exon 6 of the *WDR62* gene is predicted to cause a missense variant (p.Thr215Pro). It does not exist in various databases (dbSNP, 1000 Genomes, and Exome Variant Server). In addition, it has been predicted to be deleterious by several in silico analysis tools [SIFT damaging (0.001), PolyPhen‐2 deleterious (Naseer et al. 2017), variantTaster (Naseer et al. 2017), and REVEL (0.767)] that are damaging or probably damaging by more than one commonly used in silico prediction tool. p.Thr215Pro is located at residues highly conserved across various species (Figure 3B), and the molecular effects were further analyzed via protein modeling and stability prediction. With ΔΔG values less than −0.5 kcal/mol, the protein was predicted to have hydrogen bond interactions with surrounding residues, which significantly decreased protein stability (Figure 3C). We verified that this variant was inherited from the mother via Sanger sequencing (Figure 3A). Therefore, c.643A > C (p.Thr215Pro) was classified as “LP” on the basis of ACMG/AMP criteria: PM2_Supporting, PP1_Supporting, PP3 and PM3.

### 3.2 Molecular Effects of Candidate Variants

The molecular effects of the missense variants were further investigated through protein modeling and stability prediction. Amino acid sequence alignment of the *WDR62*‐encoded wild‐type protein revealed that residue 215 is conserved across the examined species (Figure 3B). Additionally, the variant altered hydrogen bonding with neighboring residues: while wild‐type Thr215 forms two hydrogen bonds with Arg222 and one with Trp224, the Thr215Pro substitution disrupts one bond with each of these residues (Arg222 and Trp224, respectively). This change was predicted to significantly reduce protein stability, with a ΔΔG value below −0.5 kcal/mol (Figure 3C).

### 3.3 Genotype–Phenotype Correlation

To elucidate the mechanisms underlying this phenotypic heterogeneity, we performed a systematic review of all reported *WDR62* variants in autosomal recessive primary microcephaly. A total of 44 publications were included, comprising 100 pedigrees and 217 affected individuals (Table S1). To date, more than 93 distinct *WDR62* variants have been identified in MCPH2 patients. These include 32 missense, 55 null (23 nonsense, 24 frameshift, and 8 splice‐site), 3 synonymous, and 3 intronic variants, which occur in either homozygous or compound heterozygous states and are distributed throughout the gene (Table S1; Figure 5). MRI data were available for 92 pedigrees (Table S2). Neuroimaging analysis revealed microcephaly as a conserved feature across most reported *WDR62* variant cases. Corpus callosum hypoplasia was the most common structural abnormality (35.9%, 33/92), followed by ventriculomegaly or enlarged extracerebral spaces (22.8%). Analysis of cortical malformations revealed pachygyria (47.8%), gyral simplification (33.7%), and polymicrogyria (25.0%) as the predominant anomalies. The lower‐frequency anomalies included lissencephaly (15.2%), brainstem/cerebellar anomalies (14.1%), subcortical white matter anomalies (14.1%), and schizencephaly (10.9%). Neuronal heterotopia and hippocampal abnormalities each occurred in < 10% of the cases.

**FIGURE 4:**
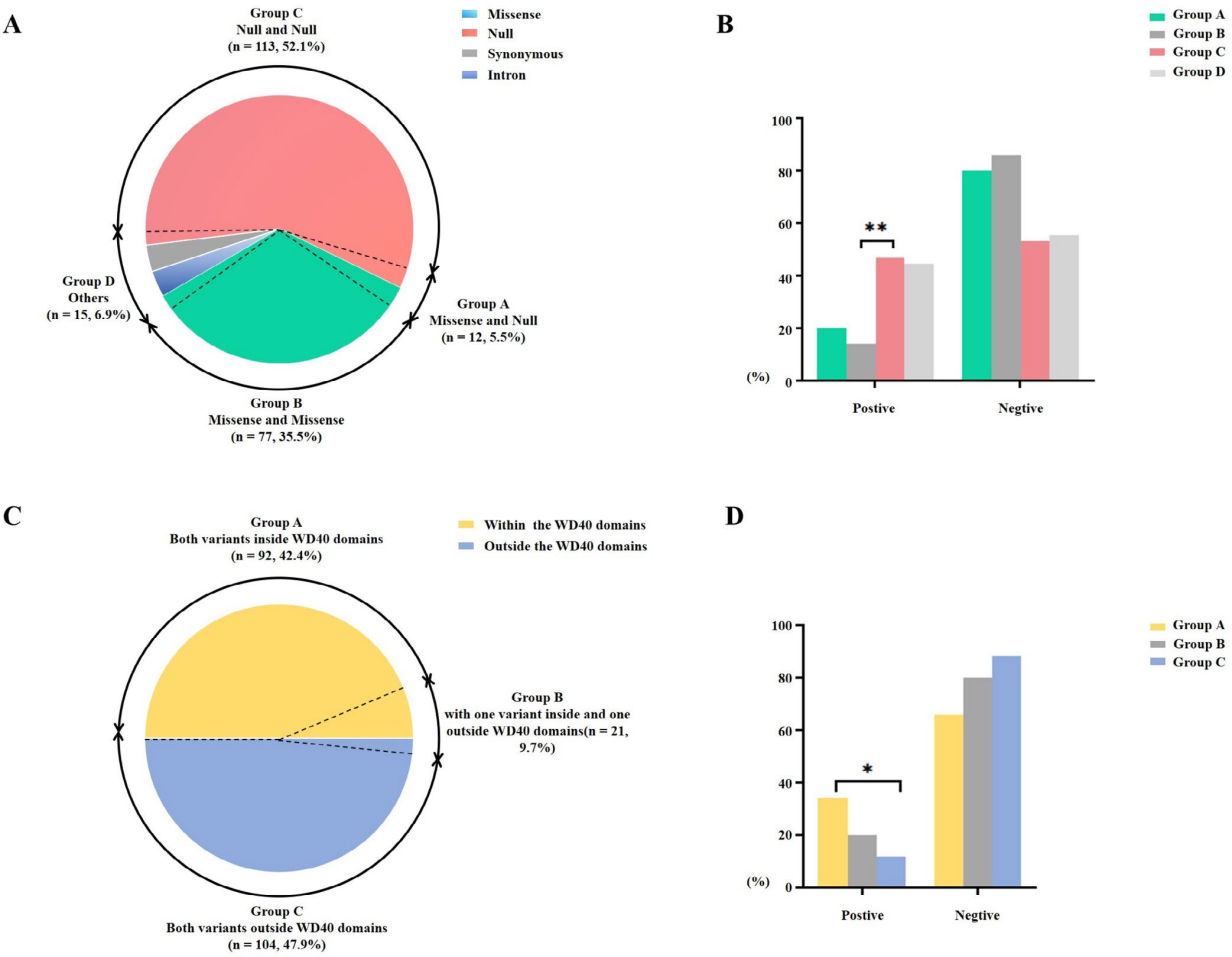
Genotype–phenotype correlation of *WDR62* variants. (A) Pie chart of the genotype distribution of *WDR62* variants. (B) The frequency of gyral simplification variants in *WDR62* for each group. Group C had a significantly higher incidence of gyral simplification than Group B (*p* = 0.006). (C) Pie chart of the location relative to the WD40 domain distribution of *WDR62* variants. (D) The frequency of ventricles or extra cerebral spaces enlargement for each group. Group A had a significantly higher incidence of ventricles or extra cerebral spaces enlargement than Group C (*p* = 0.027). Two‐tailed Fisher's exact tests were used for data processing between different phenotype groups. * *p* < 0.05, **p < 0.01.

Based on the first classification scheme, variants were divided into four groups: A (5.5%, 12/217), B (35.5%), C (52.1%), and D (6.9%) (Figure 4A). Analysis across all clinical indicators revealed that Group C had a significantly higher incidence of gyral simplification than Group B (*p* = 0.006). No significant differences were observed between Groups A and B, A and C, A and D, or C and D (Figure 4B).

**FIGURE 5:**
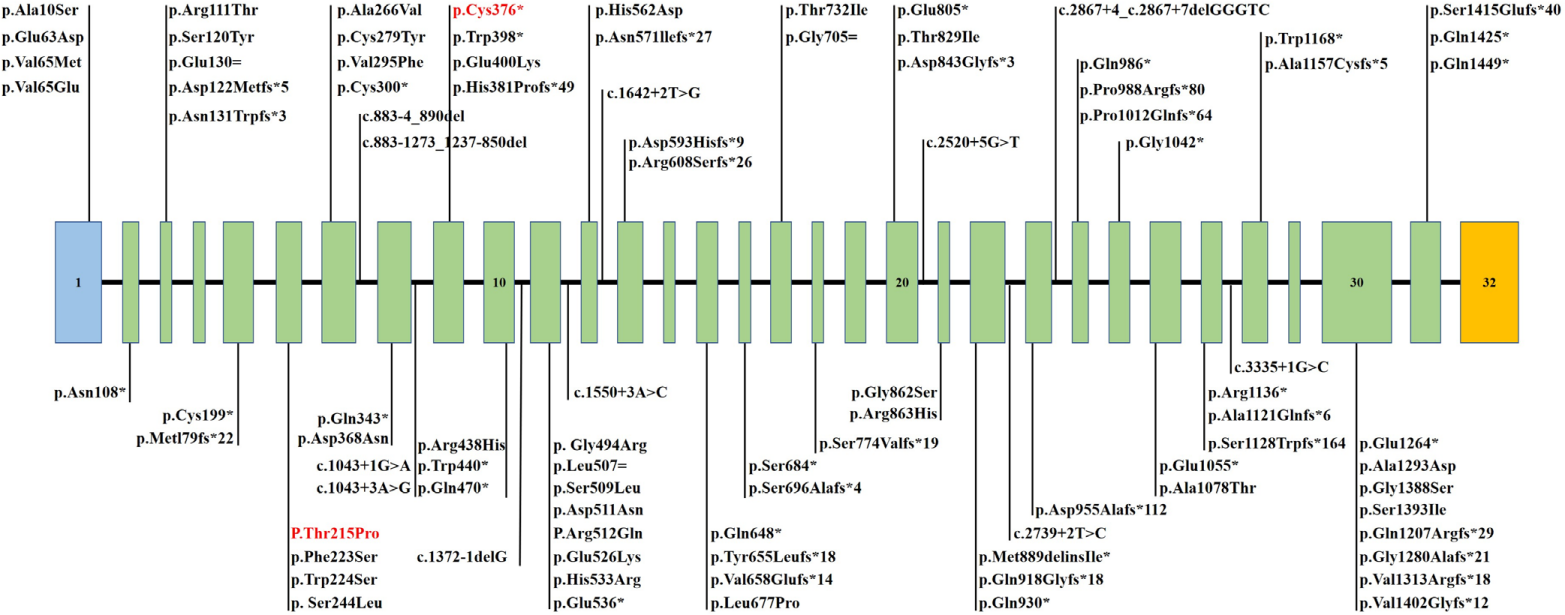
Schematic illustration of the genomic structure of human *WDR62* along with the known causal variants related to MCPH2. The results of this study are indicated by red font.

**FIGURE 6:**
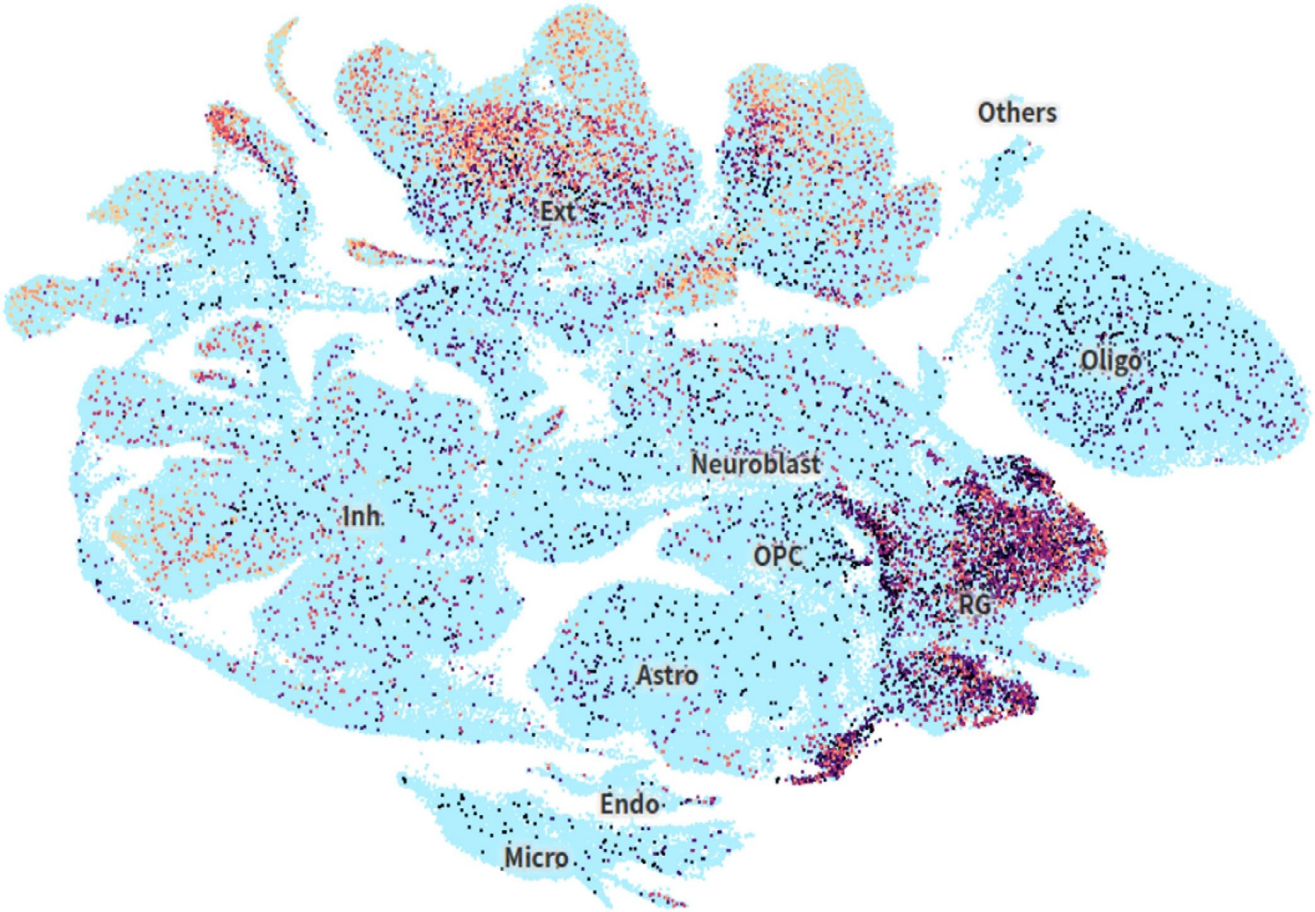
*WDR62* expression across neural cell types in the developing human brain. This figure illustrates the cell type‐specific expression pattern of *WDR62* during human brain development. The expression profile was visualized using the UCSC Cell Browser (https://cells.ucsc.edu/?ds=bts‐brain‐cell‐atlas&gene=WDR62). Astro: astrocytes; Endo: endothelial cells; Exc: excitatory neurons; Inh: inhibitory neurons; Micro: microglia; Neuroblast: neuroblasts; Oligo: oligodendrocytes; OPC: oligodendrocyte progenitor cells; Others: Other types (such as vascular smooth muscle cells, pericytes, etc.); G: radial glia.

According to the second classification scheme, variants were categorized into three groups based on their location relative to the WD40 domain: A (42.4%, 92/217), B (9.7%), and C (47.9%) (Figure 4C). In this analysis, only the incidence of ventriculomegaly or enlarged extracerebral spaces differed significantly, being higher in Group A than in Group C (*p* = 0.027). No significant differences were found between Groups A and B or between Groups B and C (Figure 4D).

## 4 Discussion

Autosomal recessive primary microcephaly (MCPH) represents a group of congenital neurodevelopmental disorders (Kaindl et al. 2010; Zaqout et al. 2017). To date, 30 distinct MCPH loci and corresponding genes (*MCPH1, WDR62, CDK5RAP2, CASC5, ASPM, CENPJ, STIL, CEP135, CEP152, ZNF335, PHC1, CDK6, CENPE, SASS6, MFSD2A, ANKLE2, CIT, WDFY3, COPB2, KIF14, NCAPD2, NCAPD3, NCAPH, NUP37, MAP11, LMNB1, LMNB2, RRP7A, PDCD6IP, and BUB1*) have been identified and characterized, establishing MCPH as a genetically heterogeneous disorder (Aslam et al. 2024). This significant genetic heterogeneity poses a substantial challenge: fetal HC measurement alone cannot definitively delineate the specific MCPH subtype. Integrating precise fetal phenotypic characterization with genetic analysis effectively overcomes this limitation. This approach provides forward‐looking information on diagnosis, prognosis, and recurrence risk. Such information is critical, enabling informed parental decision‐making and optimizing perinatal management (Plantinga et al. 2022). Fortunately, an increasing number of both imaging and molecular genetic methods are now used for prenatal diagnosis. Imaging, particularly MRI, is valuable for identifying CNS disorders, whereas molecular techniques such as trio‐WES via next‐generation sequencing (NGS) can be used to diagnose many genetic conditions (Petrovski et al. 2019). Recent studies have demonstrated that prenatal WES can detect pathogenic variants in 20%–80% of cases where conventional genetic testing (karyotype and CMA) yields normal results, with diagnostic yield significantly influenced by fetal phenotypic characteristics and detection strategies such as Trio‐WES analysis (Drury et al. 2015; Jelin and Vora 2018).

In this study, we report a family with recurrent fetal structural anomalies detected by MRI, characterized by microcephaly, ACC, and neuronal migration defects, including lobar holoprosencephaly (HPE) and lissencephaly with the agyria–pachygyria complex. The recurrence of this severe phenotypic spectrum in both fetuses can be mechanistically explained by the cell type‐specific expression of *WDR62*. Single‐cell transcriptomic analysis by Kim et al. reveals that *WDR62* is highly enriched in two key populations: radial glia, the primary neural progenitors, and early excitatory neurons (Kim et al. 2024) (Figure 6). As a centrosomal protein, *WDR62* is essential for proper mitotic progression in radial glia; its loss leads to mitotic delay, apoptosis, and depletion of the progenitor pool, directly resulting in microcephaly (Yoon et al. 2025). Concurrently, in early excitatory neurons, *WDR62* is required for centrosome‐mediated nuclear translocation during radial migration (Zombor et al. 2019). Disruption of this function underlies the co‐occurring cortical migration defects (Sgourdou et al. 2017). Thus, the combined vulnerability of these two critical cell types to *WDR62* deficiency accounts for the severe prenatal phenotype characterized by both reduced brain size and disrupted cortical architecture (Zombor et al. 2019). This mechanistic insight clarifies why compound heterozygous *WDR62* variants lead to cortical malformations that are detectable during fetal development.

Notably, the findings of previously reported cases demonstrate that identical *WDR62* variants manifest divergent neuroimaging abnormalities across unrelated families (Ruaud et al. 2022; Poulton et al. 2014; Bolat et al. 2022; Duerinckx et al. 2021) and even within single pedigrees (Yu et al. 2010; Ruaud et al. 2022; Zombor et al. 2019; Poulton et al. 2014; Bastaki et al. 2016; Murdock et al. 2011). In our study, we also observed varying degrees of phenotypic heterogeneity among individuals carrying identical compound heterozygous variants. This incomplete penetrance and variable expressivity suggest the involvement of genetic modifiers beyond a simple monogenic model. Growing evidence supports an oligogenic framework in neurodevelopmental disorders, where variants in multiple genes accumulate to exert additive or synergistic effects on key pathways, shaping clinical severity and manifestations (An et al. 2014). This framework is highly relevant to MCPH, as all known MCPH‐associated genes participate in coordinated processes like centrosome function and neural progenitor division (Bogoyevitch et al. 2012). Consequently, rare heterozygous variants in other MCPH‐related genes or mitotic regulators carried by an individual may act as modifiers, interacting with the primary pathogenic variant to exacerbate or mitigate neurodevelopmental impairment, thereby explaining the observed phenotypic diversity (Jayaraman et al. 2018). Furthermore, additional factors such as the mother's comorbid metabolic diseases during pregnancy, stochastic developmental processes in the fetus, and epigenetic modifications may also contribute to the phenotypic variability (Murdock et al. 2011). Therefore, the genotype–phenotype correlation of MCPH2 remains incompletely characterized. Consequently, prenatal detection of microcephaly via ultrasound warrants the consideration of genetic testing in differential diagnostic evaluations.

Our analysis of 92 patients revealed a key genotype–phenotype correlation: individuals harboring only null variants (Group C) had a markedly higher incidence of gyral simplification compared to those with only missense variants (Group B). This aligns with the prevailing hypothesis that null variants (such as frameshift, nonsense, and splice‐site variants), which are predicted to produce truncated proteins, contribute to more severe brain malformations. However, the correlation is not absolute. For instance, our two fetal cases each carrying one nonsense and one missense variant presented with cortical abnormalities of intermediate severity, leaning toward the severe spectrum. This observation, together with existing literature that implicates both nonsense and missense variants in severe manifestations (Nicholas et al. 2010), underscores that the genotype–phenotype relationship for MCPH2 remains incompletely defined. Consequently, when prenatal ultrasound detects microcephaly, we recommend including genetic testing in the differential diagnosis to clarify the underlying etiology.

Meanwhile, we grouped patients based on variant location relative to the WD40 protein domain. Group A (variants within the domain) showed a significantly higher incidence of enlarged ventricles/extracerebral spaces compared to Group C (variants outside the domain) (*p* = 0.027). Marie et al. demonstrated that the *N*‐terminal WD40 domains are essential for localizing the protein to spindle poles, whereas the C‐terminal region regulates spindle stability (Bogoyevitch et al. 2012). Variant in the *N*‐terminus, which abolish proper localization, results in severe spindle defects, pronounced microcephaly, and marked ventricular enlargement. In contrast, C‐terminal variants that impair regulatory function while preserving localization lead to comparatively milder phenotypic manifestations (Shohayeb et al. 2018; Slezak et al. 2021). Both variants identified in our fetuses reside within the WD40 domains; the nonsense variant is expected to cause protein truncation, while the missense variant likely disrupts protein conformation (Figure 3C). This strongly supports their pathogenicity in the observed microcephaly.

The prenatal diagnosis of microcephaly is extremely difficult, with most cases typically not detected until the mid‐to‐late gestational stages (Lerman‐Sagie et al. 2021). Both previously reported cases and our two fetal cases demonstrated frequent delays in the prenatal diagnosis of MCPH2, with most cases detected between 20 and 36 WG. Among 54 fetuses with documented birth HC measurements, 14 presented normal values at delivery despite subsequent postnatal microcephaly (Table S1), indicating that reliance on HC alone would miss nearly one‐quarter of MCPH2 diagnoses. Postnatally, previously reported cases demonstrated that DD occurred in 63.1% (137/217), ID/MR affected 52.1% of cases across mild‐to‐profound severity, and epilepsy was documented in 37.3% of cases with generalized tonic–clonic, myoclonic, or febrile seizures (Duerinckx et al. 2021; Masih et al. 2022). These neurodevelopmental manifestations remain undetectable prenatally owing to inherent fetal phenotyping limitations. Since MCPH2 is inherited in an autosomal recessive manner, affected individuals typically result from parents who are both heterozygous carriers of a pathogenic variant in the causative gene. Consequently, for such carrier couples, the recurrence risk for each pregnancy is 25%. Thus, carrier screening represents the optimal and most effective strategy for the prenatal prevention of MCPH2. This involves testing both partners prior to conception to identify heterozygous carriers of pathogenic variants associated with severe recessive genetic disorders. If both partners are identified as carriers, prenatal diagnosis can be performed via early chorionic villus sampling (CVS). Alternatively, preimplantation genetic testing for monogenic disorders (PGT‐M) can be utilized in conjunction with in vitro fertilization (IVF) to select unaffected embryos.

In summary, we report the prenatal diagnosis of MCPH2 in a family with two recurrent cases presenting microcephaly, ACC, and neuronal migration defects manifesting as lobar HPE and lissencephaly (agyria–pachygyria complex). Trio‐WES revealed compound heterozygous pathogenic variants in *WDR62*, which are consistent with the autosomal recessive inheritance pattern of MCPH2. This research expands the prenatal phenotypic spectrum of MCPH2 and enhances the use of clinical genomic databases for prenatal diagnostics. Further functional validation is necessary to elucidate the pathogenic mechanism of the *WDR62* gene in MCPH2.

## Author Contributions

Lan‐Zhen Zhang designed the study; Yan‐Fang Li and Song‐Hui Zhang completed the recruitment of the patients and the analysis of the clinical data; Yan‐Fang Li and Li Zhen completed the analysis of the genetic data; Yan‐Fang Li, Song‐Hui Zhang, and Li Zhen prepared the figures and wrote the manuscript; Lan‐Zhen Zhang revised the manuscript. All the authors have read and approved the final manuscript.

## Funding

This work was supported by the Clinical Research Fund Project of the Second Affiliated Hospital of Guangzhou Medical University (2022‐LCYJ‐YY‐05).

## Ethics Statement

This study was approved by the ethics committee of the Second Affiliated Hospital of Guangzhou Medical University (approval ethics number 2022‐hs‐60). We have conserved the parental consent documents.

## Conflicts of Interest

The authors declare no conflicts of interest.

## Supporting information


**Table S1:** Clinical characteristics of *WDR62* gene variants in a total of 217 patients with MCPH2 reported in the literature and this study.
**Table S2:** Radiographic imaging findings of WDR62 gene variants in a total of 92 patients with MCPH2 reported in the literature and this study.

## Data Availability

The data that support the findings of this study are available on request from the corresponding author. The data are not publicly available due to privacy or ethical restrictions.
