# Supplementary material for: Prenatal Diagnosis of Autosomal Recessive Primary Microcephaly Type 2 Caused by Compound Heterozygous WDR62 Variants in a Family With Two Recurrent Cases
Source: Mol Genet Genomic Med. 2026 Apr 19;14(4):e70203. doi: 10.1002/mgg3.70203 (PMC13092497; doi:10.1002/mgg3.70203)
Supplement: Supplementary file 1 — Table S1: Clinical characteristics of WDR62 gene variants in a total of 217 patients with MCPH2 reported in the literature and this study. Table S2: Radiographic imaging findings of WDR62 gene variants in a total of 92 patients with MCPH2 reported in the literature and this study. [file MGG3-14-e70203-s001.docx]

Table S1: Clinical characteristics of *WDR62* gene variants in a total of 217 patients with MCPH2 reported in the literature and this study.

| **Variation types** | **Variations**  **(n = 93)** | **Family identification  (n = 100)** | **Number of patients  (n = 217)** | **Patient identification** | **Geographic origin (n = 21)** | **Sex** | **Microcephaly detected prior birth (term in WG), n = 19, available 19/217 (8.8%)** | **OFC at birth (SD), n = 54, available 54/217 (24.9%)** | **Pregnancy complications(n = 13)** | **Age at last examination** | **OFC at last examination (SD), n = 185, available 185/217 (85.2%)** | **Epilepsy, n = 81, positive available 81/217 (37.3%)** | **DD, n = 137, positive available 137/217 (63.1%)** | **ID/MR, n = 113, positive available 113/217 (52.1%)** | **Dysmorphysm, n = 77, available 77/217 (35.5%)** | **References(n = 44)** |
| --- | --- | --- | --- | --- | --- | --- | --- | --- | --- | --- | --- | --- | --- | --- | --- | --- |
| 1. **Homozygous** | | | | | | | | | | | | | | | | |
| Missense | c.193G>A/p.Val65Met | PH-16900 | 1 | PH-16901 | Saudi Arabia | M | NA | NA | Maternal flu-like illness | 15 y 3 m | −5.3 | No | Yes | NA | Bilateral hallux valgus with pes planus and second toe overriding | Yu, 2010 |
|  | c.193G>A/p.Val65Met | PH-16900 | 1 | PH-16902 | Saudi Arabia | F | NA | NA | Poor fetal movements | 8 y 6 m | −9.8 | No | Yes | NA | No | Yu, 2010 |
|  | c.193G>A/p.Val65Met | PH-16900 | 1 | PH-16903 | Saudi Arabia | M | Yes (?) | NA | Normal pregnancy | 6 y 6 m | −9.2 | No | Yes | NA | NA | Yu, 2010 |
|  | c.193G>A/p.Val65Met | PH-16900 | 1 | PH-16907 | Saudi Arabia | F | NA | NA | Maternal flu-like illness | 12 y 3 m | Mean | Yes | Yes | NA | NA | Yu, 2010 |
|  | c.193G>A/p.Val65Met | P29436 | 3 | NA | Saudi Arabia | NA | NA | NA | NA | NA | −4 to −7 | No | Yes | Yes | Sloping forehead | Nicholas, 2010 |
|  | c.332G>C/p.Arg111Thr | MCP129 | 4 | NA | Pakistan | NA | NA | NA | NA | 8-17 y | −6 to −8 | NA | Yes | Yes | NA | Sajid Hussain, 2013 |
|  | c.332G>C/p.Arg111Thr | MCP14 | 6 | NA | Pakistan | NA | NA | NA | NA | 7-12 y | −7 to −11 | Yes | Yes | NA | NA | Ahmad, 2017 |
|  | c.359C>A/p.Ser120Tyr | NA1 | 1 | F43.1^✝^ | India | M | NA | NA | NA | 1 y 4 m | −4.5 | Yes | Yes | Yes | NA | Masih, 2022 |
|  | c.668T>C/p.Phe223Ser | NA2 | 1 | P 1 | Romania | M | No | −3.5 | No | 4 y | −6 | Yes | Yes | Yes | Sloping forehead | Zombor, 2019 |
|  | c.668T>C/p.Phe223Ser | NA2 | 1 | P 2 | Romania | F | No | −5 | Urinary tract infection, fetal bradycardia | 4 y | −6.6 | Yes | Yes | NA | Convergent squint | Zombor, 2019 |
|  | c.671G>C/p.Trp224Ser | NG 190 | 1 | NG 190-1 | NA | M | NA | NA | No | 6 y 5 m | −6.5 | Yes | Yes | Yes | Micrognathia | Bilguvar, 2010 |
|  | c.671G>C/p.Trp224Ser | NG 190 | 1 | NG 190-5 | NA | F | NA | NA | NA | 8 y 7 m | −6 | Yes | Yes | Yes | Dysconjugate gaze | Bilguvar, 2010 |
|  | c.671G>C/p.Trp224Ser | NG 190 | 1 | NG 190-6 | NA | M | NA | NA | NA | 12 y 11 m | −6 | Yes | Yes | Yes | Dysconjugate gaze | Bilguvar, 2010 |
| **Variation types** | **Variations**  **(n = 93)** | **Family identification  (n = 100)** | **Number of patients  (n = 217)** | **Patient identification** | **Geographic origin (n = 21)** | **Sex** | **Microcephaly detected prior birth (term in WG), n = 19, available 19/217 (8.8%)** | **OFC at birth (SD), n = 54, available 54/217 (24.9%)** | **Pregnancy complications(n = 13)** | **Age at last examination** | **OFC at last examination (SD), n = 185, available 185/217 (85.2%)** | **Epilepsy, n = 81, positive available 81/217 (37.3%)** | **DD, n = 137, positive available 137/217 (63.1%)** | **ID/MR, n = 113, positive available 113/217 (52.1%)** | **Dysmorphysm, n = 77, available 77/217 (35.5%)** | **References(n = 44)** |
| Missense | c.883G>T/p.Val295Phe | NA3 | 1 | P 11 | NA | M | No | −0.5 | NA | 17 y | −3 | Yes | Yes | NA | NA | Ruaud, 2021 |
|  | c.883G>T/p.Val295Phe | NA3 | 1 | P 12 | NA | F | No | −1.5 | NA | 5 y 6 m | −5.5 | No | Yes | Yes | NA | Ruaud, 2021 |
|  | c.1198G>A/p.Glu400Lys | IV | 1 | IV-7 | Spain | M | NA | NA | NA | 2 y | −4.5 | No | NA | NA | NA | Bacino, 2012 |
|  | c.1198G>A/p.Glu400Lys | IV | 1 | IV-8 | Spain | M | NA | NA | NA | 8 y | −5 | No | NA | NA | NA | Bacino, 2012 |
|  | c.1313G>A/p.Arg438His | MCP28 | 4 | NA | Pakistan | NA | NA | NA | NA | 1-36 y | −5 to −14 | NA | Yes | Yes | NA | Sajid Hussain, 2013 |
|  | c.1313G>A/p.Arg438His | MCP35 | 3 | V-2; V-3; V-5 | Pakistan | NA | NA | NA | NA | NA | −3 to −7 | No | NA | NA | NA | Kousar, 2011 |
|  | c.1313G>A/p.Arg438His | P22011a | 6 | NA | Pakistan | NA | NA | NA | NA | NA | −4 to −7 | No | Yes | Yes | Sloping forehead | Nicholas, 2010 |
|  | c.1480G>A/p.Gly494Arg | NA4 | 1 | P 7 | NA | M | No | −1 | NA | 9 y 6 m | −3 | Yes | No | Yes | NA | Ruaud, 2021 |
|  | c.1526C>T/p.Ser509Leu | NA5 | 1 | #34 | Turkey | M | NA | NA | NA | 13 y | −5 | No | NA | Yes | NA | Duerinckx, 2021 |
|  | c.1526C>T/p.Ser509Leu | NA6 | 1 | P 17 | NA | M | No | NA | NA | 13 y | −4.5 | No | Yes | NA | NA | Ruaud, 2021 |
|  | c.1531G>A/p.Asp511Asn | P22009 | 3 | NA | Pakistan | NA | NA | NA | NA | NA | −4 to −7 | No | Yes | Yes | Sloping forehead | Nicholas, 2010 |
|  | c.1531G>A/p.Asp511Asn | P22021 | 2 | NA | Pakistan | NA | NA | NA | NA | NA | −4 to −7 | No | Yes | Yes | Sloping forehead | Nicholas, 2010 |
|  | c.1531G>A/p.Asp511Asn | MCP3 | 3 | V-1; IV-3; IV-6 | Pakistan | NA | NA | NA | NA | NA | −3 to −7 | No | NA | Yes | NA | Kousar, 2011 |
|  | c.1576G>A/p.Glu526Lys | NG 537 | 1 | NG 537-1 | NA | F | NA | NA | Meconium aspiration | 14 y | −2.5 | No | Yes | Yes | Prognathism, dysconjugate gaze | Bilguvar, 2010 |
| **Variation types** | **Variations**  **(n = 93)** | **Family identification  (n = 100)** | **Number of patients  (n = 217)** | **Patient identification** | **Geographic origin (n = 21)** | **Sex** | **Microcephaly detected prior birth (term in WG), n = 19, available 19/217 (8.8%)** | **OFC at birth (SD), n = 54, available 54/217 (24.9%)** | **Pregnancy complications(n = 13)** | **Age at last examination** | **OFC at last examination (SD), n = 185, available 185/217 (85.2%)** | **Epilepsy, n = 81, positive available 81/217 (37.3%)** | **DD, n = 137, positive available 137/217 (63.1%)** | **ID/MR, n = 113, positive available 113/217 (52.1%)** | **Dysmorphysm, n = 77, available 77/217 (35.5%)** | **References(n = 44)** |
| Missense | c.1598A>G/ p.His533Arg | NA7 | 1 | III.1 | NA | M | NA | -1 | No | 27 y | NA | Yes | Yes | Yes | NA | Hajar, 2022 |
|  | c.1598A>G/ p.His533Arg | NA7 | 1 | III.2 | NA | M | NA | -1 | No | 9 y | NA | Yes | Yes | Yes | NA | Hajar, 2022 |
|  | c.2030T>C/p.Leu677Pro | NA8 | 1 | P 8 | NA | F | No | −3.5 | NA | 16 y 6 m | −7 | Yes | Yes | NA | NA | Ruaud, 2021 |
|  | c.2195C>T/p.Thr732Ile | Fam.4 | 1 | V-4 | Pakistan | F | NA | NA | NA | 8 y | −11 | No | Yes | Yes | NA | Rasool, 2020 |
|  | c.2195C>T/p.Thr732Ile | Fam.4 | 1 | V-6 | Pakistan | F | NA | NA | NA | 18 y | −12.5 | No | NA | Yes | NA | Rasool, 2020 |
|  | c.2195C>T/p.Thr732Ile | Fam.4 | 1 | VI-1 | Pakistan | M | NA | NA | NA | 8 y | −9.5 | No | NA | Yes | NA | Rasool, 2020 |
|  | c.2195C>T/p.Thr732Ile | Fam.4 | 1 | VI-3 | Pakistan | M | NA | NA | NA | 11 y | −10 | No | Yes | Yes | NA | Rasool, 2020 |
|  | c.2195C>T/p.Thr732Ile | Fam.4 | 1 | VI-4 | Pakistan | F | NA | NA | NA | 2 y | −7 | No | NA | Yes | NA | Rasool, 2020 |
|  | c.2588G>A/p.Arg863His | NA9 | 1 | P 3 | Tunisia | F | No | −3 | Maternal diabetes | 6 y | −4 | No | Yes | NA | Sloping forehead, small chin, prominent nose, upward eyelid slant | Poulton, 2014 |
|  | c.2588G>A/p.Arg863His | NA10 | 1 | P 13 | NA | F | NA | NA | NA | 21 y | −2.5 | Yes | Yes | NA | NA | Ruaud, 2021 |
|  | c.2588G>A/p.Arg863His | NA10 | 1 | P 14 | NA | F | No | −0.5 | NA | 6 y | −2.5 | Yes | Yes | Yes | Strabismus | Ruaud, 2021 |
|  | c.3232G>A/p.Ala1078Thr | MCP91 | 5 | NA | Pakistan | NA | NA | NA | NA | NA | −4 to −7 | No | Yes | Yes | Sloping forehead | Nicholas, 2010 |
|  | c.3878C>A/p.Ala1293Asp | IV | 1 | IV-1 | Saudi Arabia | F | NA | NA | NA | 23 y | −5 | Yes | Yes | Yes | NA | Naseer, 2017 |
|  | c.3878C>A/p.Ala1293Asp | IV | 1 | IV-4 | Saudi Arabia | M | NA | NA | NA | 7 y | −5 | Yes | Yes | Yes | NA | Naseer, 2017 |
| **Variation types** | **Variations**  **(n = 93)** | **Family identification  (n = 100)** | **Number of patients  (n = 217)** | **Patient identification** | **Geographic origin (n = 21)** | **Sex** | **Microcephaly detected prior birth (term in WG), n = 19, available 19/217 (8.8%)** | **OFC at birth (SD), n = 54, available 54/217 (24.9%)** | **Pregnancy complications(n = 13)** | **Age at last examination** | **OFC at last examination (SD), n = 185, available 185/217 (85.2%)** | **Epilepsy, n = 81, positive available 81/217 (37.3%)** | **DD, n = 137, positive available 137/217 (63.1%)** | **ID/MR, n = 113, positive available 113/217 (52.1%)** | **Dysmorphysm, n = 77, available 77/217 (35.5%)** | **References(n = 44)** |
| Nonsense | c.321dupT/p.Asn108* | 26 | 1 | A33021 | China | NA | Yes (?) | NA | NA | NA | NA | Yes | Yes | NA | NA | Wang, 2023 |
|  | c.597_599del/p.Cys199* | NA11 | 1 | P 10 | NA | F | Yes(20) | −3 | NA | 15 y | −5 | No | Yes | NA | NA | Ruaud, 2021 |
|  | c.900C>A/p.Cys300* | IIS-43 | 1 | IV-1 | India | M | NA | NA | NA | 11 y | −8.5 | Yes | Yes | Yes | NA | Bhat, 2011 |
|  | c.900C>A/p.Cys300* | IIS-43 | 1 | IV-2 | India | F | NA | NA | NA | 7 y | −10 | NA | Yes | Yes | NA | Bhat, 2011 |
|  | c.1027C>T/p.Gln343* | V | 1 | V.3 | Morocco | M | Yes (?) | −3 | No | 11 y | −5.5 | No | Yes | Yes | NA | Jaouad, 2018 |
|  | c.1027C>T/p.Gln343* | V | 1 | V.4 | Morocco | F | No | −3 | No | 9 y | −7 | No | Yes | Yes | NA | Jaouad, 2018 |
|  | c.1194G>A/p.Trp398* | 21 | 1 | IV-1 | Pakistan | M | NA | NA | NA | 15 y | −5 | No | NA | Yes | NA | Rasool, 2020 |
|  | c.1194G>A/p.Trp398* | 21 | 1 | IV-2 | Pakistan | M | NA | NA | NA | 24 y | −5.5 | Yes | Yes | Yes | NA | Rasool, 2020 |
|  | c.1194G>A/p.Trp398* | MCP1 | 4 | NA | Pakistan | M | NA | NA | NA | 8-17 y | −6 to −8 | NA | NA | NA | NA | Sajid Hussain, 2013 |
|  | c.1319G>A/p.Trp440* | 9506900 | 1 | 202 | Iran | F | NA | NA | NA | 6 y | NA | Yes | Yes | NA | Strabismus | Abolhassani, 2024 |
|  | c.1408C>T/p.Gln470* | NG 294 | 1 | NG 294-1 | NA | M | NA | NA | Preterm birth (32 weeks) | 14 y 6 m | NA | No | NA | Yes | Arachnodactly, bow leggedness | Bilguvar, 2010 |
|  | c.1576G>T/p.Glu526* | NG 30 | 1 | NG 30-1 | NA | F | NA | NA | NA | 7 y | −5.5 | Yes | Yes | Yes | NA | Bilguvar, 2010 |
|  | c.1605dupT/p.Glu536* | NA12 | 1 | P 1 | Turkey | F | Yes (34) | −3.5 | NA | 2 y | −9 | Yes | Yes | Yes | Hypertelorism, upslanted palpebral fissures, large ears, broad thumbs, short neck | Poulton, 2014 |
|  | c.1605dupT/p.Glu536* | F2 | 2 | Case 3 | Turkey | M | NA | −2.17 | NA | 8 y | −5.4 | Yes | Yes | NA | Sloping forehead | Bolat, 2022 |
|  | c.1605dupT/p.Glu536* | F3 | 1 | case 5 | Turkey | F | NA | −3.62 | NA | 2 y | −4.0 | Yes | Yes | NA | No | Bolat, 2022 |
| **Variation types** | **Variations**  **(n = 93)** | **Family identification  (n = 100)** | **Number of patients  (n = 217)** | **Patient identification** | **Geographic origin (n = 21)** | **Sex** | **Microcephaly detected prior birth (term in WG), n = 19, available 19/217 (8.8%)** | **OFC at birth (SD), n = 54, available 54/217 (24.9%)** | **Pregnancy complications(n = 13)** | **Age at last examination** | **OFC at last examination (SD), n = 185, available 185/217 (85.2%)** | **Epilepsy, n = 81, positive available 81/217 (37.3%)** | **DD, n = 137, positive available 137/217 (63.1%)** | **ID/MR, n = 113, positive available 113/217 (52.1%)** | **Dysmorphysm, n = 77, available 77/217 (35.5%)** | **References(n = 44)** |
| Nonsense | c.1605dupT/p.Glu536* | F4 | 1 | case 6 | Turkey | M | NA | −2.54 | NA | 16 y | −6.3 | No | Yes | NA | Prominent forehead | Bolat, 2022 |
|  | c.1605dupT/p.Glu536* | NA13 | 1 | P6 | NA | NA | NA | −2.7 | NA | NA | NA | No | Yes | Yes | NA | Poulton, 2014 |
|  | c.1942C>T/p.Gln648* | MCP67 | 1 | IV-5 | Pakistan | M | NA | NA | NA | NA | −3 to −7 | No | NA | NA | No | Kousar, 2011 |
|  | c.1942C>T/p.Gln648* | MCP67 | 1 | IV-6 | Pakistan | M | NA | NA | NA | NA | NA | No | NA | NA | No | Kousar, 2011 |
|  | c.2667_2668delGAinsTT/p.Met889delinsIle* | M-F1 | 2 | NA | Pakistan | NA | NA | NA | NA | 6 m-59 y | −9 to –10 | Yes | NA | NA | NA | Wang, 2017 |
|  | c.2956C>T/p.Gln986* | F5 | 1 | 7 | NA | F | NA | −3.2 | NA | 5 y | −5 | Yes | Yes | NA | No | Bolat, 2022 |
|  | c.3406C>T/p.Arg1136* | S228 | 1 | II:1 | Turkey | M | NA | NA | NA | 1 y | NA | NA | Yes | NA | Bilateral epicanthus | McSherry, 2018 |
|  | c.3503G>A/p.Trp1168* | MCP59 | 3 | NA | Pakistan | M | NA | NA | NA | 16-18 y | −9 to −11 | Yes | Yes | NA | NA | Sajid Hussain, 2013 |
|  | c.3790G>T/p.Glu1264* | NA14 | 1 | P 9 | NA | F | No | −1 | NA | 5 y | −5 | Yes | Yes | Yes | NA | Ruaud, 2021 |
|  | c.4345C>T/p.Gln1449* | NA15 | 1 | P 5 | NA | M | Yes (?) | −2.5 | NA | 13 y | −4 | Yes | Yes | NA | NA | Ruaud, 2021 |
| Synonymous | c.390G>A/p.Glu130= | IV | 1 | IV-4 | Sudan | F | No | −3 | NA | 9 y | −6.5 | Yes | Yes | NA | Esotropia | Bastaki, 2016 |
|  | c.390G>A/p.Glu130= | IV | 1 | IV-5 | Sudan | M | Yes (?) | −3 | No | 11 y | −5 | NA | Yes | NA | NA | Bastaki, 2016 |
|  | c.2115C>G/p.Gly705= | M196 | 1 | IV:1 | Iran | F | NA | NA | NA | 20 y | NA | Yes | NA | Yes | NA | Hu, 2014 |
|  | c.2115C>G/p.Gly705= | M196 | 1 | IV:4 | Iran | M | NA | NA | NA | 16 y | NA | Yes | NA | Yes | NA | Hu, 2014 |
| Frameshift | c.363delT/p.Asp122Metfs*5 | LIS-900 | 1 | LIS-901 | Mexico | M | NA | −1.5 | No | 7 y | NA | No | Yes | NA | Soping forehead | Yu, 2010 |
|  | c.363delT/p.Asp122Metfs*5 | LIS-900 | 1 | LIS-902 | Mexico | F | NA | NA | Difficult delivery with forceps | 12 y | NA | Yes | Yes | NA | Soping forehead | Yu, 2010 |
| **Variation types** | **Variations**  **(n = 93)** | **Family identification  (n = 100)** | **Number of patients  (n = 217)** | **Patient identification** | **Geographic origin (n = 21)** | **Sex** | **Microcephaly detected prior birth (term in WG), n = 19, available 19/217 (8.8%)** | **OFC at birth (SD), n = 54, available 54/217 (24.9%)** | **Pregnancy complications(n = 13)** | **Age at last examination** | **OFC at last examination (SD), n = 185, available 185/217 (85.2%)** | **Epilepsy, n = 81, positive available 81/217 (37.3%)** | **DD, n = 137, positive available 137/217 (63.1%)** | **ID/MR, n = 113, positive available 113/217 (52.1%)** | **Dysmorphysm, n = 77, available 77/217 (35.5%)** | **References(n = 44)** |
| Frameshift | c.363delT/p.Asp122Metfs*5 | LIS-900 | 1 | LIS-903 | Mexico | F | NA | NA | Premature rupture of membranes | 3 y | −5.4 | No | Yes | NA | Soping forehead | Yu, 2010 |
|  | c.384_385delAG/p.Asn131Trpfs*3 | F6 | 1 | 8 | Turkey | F | NA | −2.5 | NA | 10 y | −5.6 | Yes | Yes | NA | No | Bolat, 2022 |
|  | c.535dup/p.Met179Asnfs*22 | IIS-36 | 1 | IV-2 | India | F | NA | NA | NA | 6 y | −8.5 | Yes | Yes | NA | NA | Bhat, 2011 |
|  | c.535dup/p.Met179Asnfs*22 | IIS-36 | 1 | IV-3 | India | M | NA | NA | NA | 5 y | −5.5 | No | NA | Yes | NA | Bhat, 2011 |
|  | c.1143delC/p.His381Glnfs*49 | V | 1 | V-2 | Pakistan | M | NA | NA | NA | 7 y | −7.5 | No | Yes | NA | NA | Memon, 2013 |
|  | c.1143delC/p.His381Glnfs*49 | V | 1 | V-3 | Pakistan | F | NA | NA | NA | 9 y | −4.5 | Yes | Yes | NA | NA | Memon, 2013 |
|  | c.1821dupT/p.Arg608Serfs*26 | II | 1 | II-1 | Quebec | F | NA | NA | NA | 59 y | −4.5 | Yes | NA | Yes | Sloping forehead | McDonell, 2014 |
|  | c.1821dupT/p.Arg608Serfs*26 | II | 1 | II-2 | Quebec | M | NA | NA | NA | 56 y | −2.5 | Yes | NA | Yes | Sloping forehead | McDonell, 2014 |
|  | c.1821dupT/p.Arg608Serfs*26 | II | 1 | II-3 | Quebec | M | NA | NA | NA | 55 y | −3.5 | Yes | NA | Yes | Sloping forehead | McDonell, 2014 |
|  | c.1821dupT/p.Arg608Serfs*26 | II | 1 | II-4 | Quebec | F | NA | NA | NA | 53 y | −5 | Yes | NA | Yes | Sloping forehead | McDonell, 2014 |
|  | c.1973_1974del/p.Val658Glufs*14 | 13 | 1 | IV-1 | Iraq | F | NA | NA | NA | 27 y | −6 | Yes | NA | Yes | NA | Kvarnung, 2018 |
|  | c.1973_1974del/p.Val658Glufs*14 | 13 | 1 | IV-2 | Iraq | F | NA | NA | NA | 28 y | −6 | Yes | NA | Yes | NA | Kvarnung, 2018 |
|  | c.1973_1974del/p.Val658Glufs*14 | 13 | 1 | IV-3 | Iraq | M | NA | NA | NA | 32 y | −6 | No | NA | Yes | NA | Kvarnung, 2018 |
|  | c.2527dupG/p.Asp843Glyfs*3 | MCP1 | 1 | MCP1-2 | Pakistan | M | NA | NA | NA | 4 y 6 m | −9.5 | NA | NA | NA | NA | Rupp, 2014 |
|  | c.2527dupG/p.Asp843Glyfs*3 | MCP1 | 1 | MCP1-5 | Pakistan | M | NA | NA | NA | 2 y | −9.5 | NA | NA | NA | NA | Rupp, 2014 |
| **Variation types** | **Variations**  **(n = 93)** | **Family identification  (n = 100)** | **Number of patients  (n = 217)** | **Patient identification** | **Geographic origin (n = 21)** | **Sex** | **Microcephaly detected prior birth (term in WG), n = 19, available 19/217 (8.8%)** | **OFC at birth (SD), n = 54, available 54/217 (24.9%)** | **Pregnancy complications(n = 13)** | **Age at last examination** | **OFC at last examination (SD), n = 185, available 185/217 (85.2%)** | **Epilepsy, n = 81, positive available 81/217 (37.3%)** | **DD, n = 137, positive available 137/217 (63.1%)** | **ID/MR, n = 113, positive available 113/217 (52.1%)** | **Dysmorphysm, n = 77, available 77/217 (35.5%)** | **References(n = 44)** |
| Frameshift | c.2527dupG/p.Asp843Glyfs*3 | MCP1 | 1 | MCP1-6 | Pakistan | M | NA | NA | No | 25 y | −9.5 | NA | No | No | No | Rupp, 2014 |
|  | c.2864_2867delACAG/p.Asp955Alafs*112 | NG1406 | 1 | NG1406-1 | Turkey | M | NA | NA | NA | 8 y | −6 | NA | Yes | Yes | Large auricles | Sgourdou, 2017 |
|  | c.2864_2867delACAG/p.Asp955Alafs*112 | NG1406 | 1 | NG1406-2 | Turkey | M | NA | −2 | NA | 3 y 6 m | −6 | NA | Yes | Yes | Hypoplastic forehead, large auricles | Sgourdou, 2017 |
|  | c.2864_2867delACAG/p.Asp955Alafs*112 | NA16 | 1 | P 2 | NA | M | NA | NA | NA | 13 y | NA | Yes | Yes | NA | NA | Poulton, 2014 |
|  | c.3361delG/p.Ala1121Glnfs*6 | 10 | 1 | V-1 | Pakistan | M | NA | NA | NA | NA | NA | NA | NA | NA | NA | Rasool, 2020 |
|  | c.3361delG/p.Ala1121Glnfs*6 | 10 | 1 | V-3 | Pakistan | F | NA | NA | NA | NA | NA | NA | NA | NA | NA | Rasool, 2020 |
|  | c.3361delG/p.Ala1121Glnfs*6 | 10 | 1 | VI-1 | Pakistan | M | NA | NA | NA | 16 y | −6.8 | No | Yes | Yes | NA | Rasool, 2020 |
|  | c.3361delG/p.Ala1121Glnfs*6 | 10 | 1 | VI-5 | Pakistan | M | NA | NA | NA | 7 y | −7 | No | NA | Yes | NA | Rasool, 2020 |
|  | c.3361delG/p.Ala1121Glnfs*6 | 10 | 1 | VI-6 | Pakistan | F | NA | NA | NA | 2 y | −9.8 | No | NA | Yes | NA | Rasool, 2020 |
|  | c.3361delG/p.Ala1121Glnfs*6 | MCP87 | 3 | NA | Pakistan | M | NA | NA | NA | 15-18 y | −10 to −11 | NA | NA | NA | NA | Sajid Hussain, 2013 |
|  | c.3839_3855delGCCAAGAGCCTGCCCTG/p.Gly1280Alafs*21 | MC-1600 | 1 | MC-1601 | Turkey | M | NA | NA | NA | 4 y | NA | Yes | Yes | NA | NA | Yu, 2010 |
|  | c.3839_3855delGCCAAGAGCCTGCCCTG/p.Gly1280Alafs*21 | NG 339 | 1 | NG 339-1 | NA | F | NA | NA | NA | 3 m | −4 | No | Yes | Yes | NA | Bilguvar, 2010 |
|  | c.3936dupC/p.Val1313Argfs*18 | PC12 | 1 | NA | Belgium | F | NA | −2 | NA | 10 y | −5 | No | Yes | Yes | NA | Nicholas, 2010 |
|  | c.3936dupC/p.Val1313Argfs*18 | MCP26 | 3 | IV-3; IV-6; IV-8 | Pakistan | NA | NA | NA | NA | NA | −3 to −7 | No | NA | NA | No | Kousar, 2011 |
|  | c.3936dupC/p.Val1313Argfs*18 | LIS-2600 | 1 | LIS-2601 | Turkey | NA | Yes (27) | NA | No | NA | NA | NA | NA | NA | NA | Yu, 2010 |
| **Variation types** | **Variations**  **(n = 93)** | **Family identification  (n = 100)** | **Number of patients  (n = 217)** | **Patient identification** | **Geographic origin (n = 21)** | **Sex** | **Microcephaly detected prior birth (term in WG), n = 19, available 19/217 (8.8%)** | **OFC at birth (SD), n = 54, available 54/217 (24.9%)** | **Pregnancy complications(n = 13)** | **Age at last examination** | **OFC at last examination (SD), n = 185, available 185/217 (85.2%)** | **Epilepsy, n = 81, positive available 81/217 (37.3%)** | **DD, n = 137, positive available 137/217 (63.1%)** | **ID/MR, n = 113, positive available 113/217 (52.1%)** | **Dysmorphysm, n = 77, available 77/217 (35.5%)** | **References(n = 44)** |
| Frameshift | c.3936dupC/p.Val1313Argfs*18 | LIS-2600 | 1 | LIS-2602 | Turkey | M | Yes (25) | −3 | No | 2 y 3 m | −3 | NA | Yes | NA | Low sloping forehead, prominent occiput,broad and prominent nasal bridge. | Yu, 2010 |
|  | c.3936dupC/p.Val1313Argfs*18 | 8 | 1 | IV-1 | Pakistan | F | NA | NA | NA | 30 y | −10.7 | No | NA | Yes | NA | Rasool, 2020 |
|  | c.3936dupC/p.Val1313Argfs*18 | 8 | 1 | IV-3 | Pakistan | M | NA | NA | NA | 26 y | −8 | No | NA | Yes | NA | Rasool, 2020 |
|  | c.3936dupC/p.Val1313Argfs*18 | 8 | 1 | IV-7 | Pakistan | M | NA | NA | NA | 22 y | −6.8 | No | NA | NA | NA | Rasool, 2020 |
|  | c.3936dupC/p.Val1313Argfs*18 | Family B | 1 | IV-2 | Pakistan | F | NA | NA | No | 10 y | −10.9 | Yes | Yes | Yes | Squint eyes, sloping forehead, narrow nasal tip | Aslam, 2024 |
|  | c.3936dupC/p.Val1313Argfs*18 | Family B | 1 | IV-3 | Pakistan | M | NA | NA | No | 8 y | −8.3 | Yes | Yes | Yes | Squint eyes, sloping forehead, narrow nasal tip | Aslam, 2024 |
|  | c.3936dupC/p.Val1313Argfs*18 | NA17 | 1 | #38 | Belgium | F | NA | −2.5 | NA | 9 y | −8 | No | NA | Yes | NA | Duerinckx, 2021 |
|  | c.3936dupC/p.Val1313Argfs*18 | NA18 | 1 | P 3 | NA | F | Yes (?) | −2.5 | NA | 12 y 6 m | −7 | No | Yes | NA | NA | Ruaud, 2021 |
|  | c.4205_4208delTGCC/p.Val1402Glyfs*12 | NG 26 | 1 | NG 26-1 | NA | F | NA | NA | NA | 2 y 3 m | −8 | No | NA | Yes | Micrognathia, bulbous nose | Bilguvar, 2010 |
|  | c.4205_4208delTGCC/p.Val1402Glyfs*12 | NG 891 | 1 | NG 891-1 | NA | M | NA | NA | NA | 1 y 8 m | −4.5 | No | Yes | Yes | No | Bilguvar, 2010 |
|  | c.4241dupT/p.Ser1415Glufs*40 | P22016a | 9 | NA | Pakistan | NA | NA | NA | NA | NA | −4 to −7 | No | Yes | Yes | Sloping forehead | Nicholas, 2010 |
|  | c.4241dupT/p.Ser1415Glufs*40 | 9 | 1 | IV-3 | Pakistan | M | NA | NA | NA | NA | NA | NA | NA | NA | NA | Rasool, 2020 |
|  | c.4241dupT/p.Ser1415Glufs*40 | 9 | 1 | V-2 | Pakistan | F | NA | NA | NA | NA | NA | NA | NA | NA | NA | Rasool, 2020 |
|  | c.4241dupT/p.Ser1415Glufs*40 | 9 | 1 | V-3 | Pakistan | M | NA | NA | NA | NA | NA | NA | NA | NA | NA | Rasool, 2020 |
|  | c.4241dupT/p.Ser1415Glufs*40 | 9 | 1 | V-4 | Pakistan | F | NA | NA | NA | NA | NA | NA | NA | NA | NA | Rasool, 2020 |
| **Variation types** | **Variations**  **(n = 93)** | **Family identification  (n = 100)** | **Number of patients  (n = 217)** | **Patient identification** | **Geographic origin (n = 21)** | **Sex** | **Microcephaly detected prior birth (term in WG), n = 19, available 19/217 (8.8%)** | **OFC at birth (SD), n = 54, available 54/217 (24.9%)** | **Pregnancy complications(n = 13)** | **Age at last examination** | **OFC at last examination (SD), n = 185, available 185/217 (85.2%)** | **Epilepsy, n = 81, positive available 81/217 (37.3%)** | **DD, n = 137, positive available 137/217 (63.1%)** | **ID/MR, n = 113, positive available 113/217 (52.1%)** | **Dysmorphysm, n = 77, available 77/217 (35.5%)** | **References(n = 44)** |
| Frameshift | c.4241dupT/p.Ser1415Glufs*40 | 9 | 1 | V-5 | Pakistan | F | NA | NA | NA | NA | NA | NA | NA | NA | NA | Rasool, 2020 |
|  | c.4241dupT/p.Ser1415Glufs*40 | 9 | 1 | V-7 | Pakistan | M | NA | NA | NA | 29 y | −8 | Yes | Yes | Yes | NA | Rasool, 2020 |
|  | c.4241dupT/p.Ser1415Glufs*40 | 9 | 1 | V-10 | Pakistan | M | NA | NA | NA | 8 y | −8 | No | NA | Yes | NA | Rasool, 2020 |
| Splicesite | c.1043+1G>A/- | LIS-2500 | 1 | LIS-2501 | Turkey | F | NA | −2.6 | NA | 5 m | −5.3 | No | Yes | NA | NA | Yu, 2010 |
|  | c.1043+3A>G/- | NA19 | 1 | #35 | Belgium | M | No | 0.5 | NA | 13 y | −2.5 | Yes | NA | No | NA | Duerinckx, 2021 |
|  | c.2520+5G>T/- | M-F43 | 2 | NA | Pakistan | F | NA | NA | NA | 6 m-59 y | −13 to –14 | Yes | NA | NA | NA | Wang, 2017 |
|  | c.2739+2T>C/- | NA20 | 1 | P 1 | NA | M | No | −2 | NA | 13 y 3 m | −2.5 | Yes | Yes | Yes | Strabismus, hypermetropia, astigmatism | Ruaud, 2021 |
|  | c.3335+1G>C/- | NA21 | 1 | NA | Italy | M | NA | NA | NA | 9 y | −2 | Yes | Yes | Yes | Narrow forehead, prominent nose, large ears, short nasal bridge, anteverted nostrils, full lips, microretrognathia, and dental dysplasia. | Nardello, 2018 |
|  | c.1372-1delG/- | Family A | 1 | VI-8 | Pakistan | M | NA | NA | No | 7 y | −10.5 | No | Yes | Yes | Squint eyes | Aslam, 2024 |
|  | c.1372-1delG/- | Family A | 1 | VI-9 | Pakistan | M | NA | NA | No | 5 y | −7.7 | No | Yes | Yes | Squint eyes | Aslam, 2024 |
| Intron | c.2867+4_c.2867+7delGGGTC | MC-1400 | 1 | MC-1403 | Turkey | F | Yes (28) | NA | NA | 1 y | −8.5 | No | Yes | NA | No | Yu, 2010 |
|  | c.883-1273_1237-850del | M-F17 | 5 | NA | Pakistan | F | NA | NA | NA | 6 m-59 y | −5.6 to –11 | Yes | NA | NA | NA | Wang, 2017 |
| **Variation types** | **Variations**  **(n = 93)** | **Family identification  (n = 100)** | **Number of patients  (n = 217)** | **Patient identification** | **Geographic origin (n = 21)** | **Sex** | **Microcephaly detected prior birth (term in WG), n = 19, available 19/217 (8.8%)** | **OFC at birth (SD), n = 54, available 54/217 (24.9%)** | **Pregnancy complications(n = 13)** | **Age at last examination** | **OFC at last examination (SD), n = 185, available 185/217 (85.2%)** | **Epilepsy, n = 81, positive available 81/217 (37.3%)** | **DD, n = 137, positive available 137/217 (63.1%)** | **ID/MR, n = 113, positive available 113/217 (52.1%)** | **Dysmorphysm, n = 77, available 77/217 (35.5%)** | **References(n = 44)** |
| 1. **compound heterozygous** | | | | | | | | | | | | | | | | |
| Missense  Missense | c.28G>T/p.Ala10Ser c.189G>T/p.Glu63Asp | NA22 | 1 | NA | China | F | NA | NA | NA | 5 y | −6 | No | Yes | NA | A wide and low bridge of the nose, thick lips, broad eye distance, sloping forehead, high palate-maxillary arch and low-set and large ears. | Banerjee, 2016 |
|  | c.643A>C/p.Thr215Pro c.1535G>A/p.Arg512Gln | NA23 | 1 | CP_051_1 | China | F | NA | NA | NA | 3 y 10 m | −5.2 | NA | NA | Yes | NA | Li, 2022 |
|  | c.797C>T/p.Ala266Val c.1102G>A/p.Asp368Asn | IV | 1 | IV-1 | Sudan | F | NA | NA | NA | 11 y | −3.8 | No | Yes | NA | Proptosis | Naseer, 2019 |
|  | c.797C>T/p.Ala266Val c.1102G>A/p.Asp368Asn | IV | 1 | IV-2 | Sudan | F | NA | NA | NA | 9 y | −3.2 | No | Yes | NA | No | Naseer, 2019 |
|  | c.836G>A/p.Cys279Tyr c.1480G>A/p.Gly494Arg | 25 | 1 | CS19 | Belgium | F | NA | NA | NA | NA | NA | Yes | NA | NA | NA | Stutterd, 2021 |
|  | c.836G>A/p.Cys279Tyr c.1480G>A/p.Gly494Arg | 26 | 1 | CS20 | Belgium | F | NA | NA | NA | NA | NA | No | NA | NA | NA | Stutterd, 2021 |
|  | c.4162G>A/p.Gly1388Ser c.4178G>T/p.Ser1393Ile | NA24 | 1 | P 6 | NA | F | No | −1.5 | NA | 7 y | −3 | Yes | No | Yes | NA | Ruaud, 2021 |
| Missense  Nonsense | c.194T>A/p.Val65Glu c.4273C>T/p.Gln1425* | b | 1 | Case 2 | Poland | M | No | −2 | No | 20 m | > −2 | No | Yes | No | Sloping forehead, upslanted palpebral fissures | Slezak, 2021 |
|  | c.643A>C/p.Thr215Pro c.1128C>A/p.Cys376* | NA25 | 1 | Ⅱ:2 | China | F | Yes (29) | −3 | No | NA | NA | NA | NA | NA | NA | This stdy |
|  | c.643A>C/p.Thr215Pro c.1128C>A/p.Cys376* | NA25 | 1 | Ⅱ:3 | China | M | Yes (26) | −3 | No | NA | NA | NA | NA | NA | NA | This stdy |
|  | c.731C>T/p.Ser244Leu c.2413G>T/p.Glu805* | II | 1 | II-2 | Japan | M | No | −1.5 | No | 4 y 11 m | −2.7 | No | NA | Yes | NA | Miyamoto, 2017 |
|  | c.731C>T/p.Ser244Leu c.2413G>T/p.Glu805* | II | 1 | II-3 | Japan | F | Yes (?) | −2.8 | No | 11 m | −3.9 | No | Yes | NA | NA | Miyamoto, 2017 |
| **Variation types** | **Variations**  **(n = 93)** | **Family identification  (n = 100)** | **Number of patients  (n = 217)** | **Patient identification** | **Geographic origin (n = 21)** | **Sex** | **Microcephaly detected prior birth (term in WG), n = 19, available 19/217 (8.8%)** | **OFC at birth (SD), n = 54, available 54/217 (24.9%)** | **Pregnancy complications(n = 13)** | **Age at last examination** | **OFC at last examination (SD), n = 185, available 185/217 (85.2%)** | **Epilepsy, n = 81, positive available 81/217 (37.3%)** | **DD, n = 137, positive available 137/217 (63.1%)** | **ID/MR, n = 113, positive available 113/217 (52.1%)** | **Dysmorphysm, n = 77, available 77/217 (35.5%)** | **References(n = 44)** |
| Missense  Nonsense | c.1531G>A/p.Asp511Asn c.4345C>T/p.Gln1449* | NA26 | 1 | #32 | Caucasian | M | NA | −2 | NA | 26 y | −5 | Yes | NA | Yes | NA | Duerinckx, 2021 |
| Missense  Frameshift | c.1313G>A/p.Arg438His c.2864_2867delACAG/p.Asp955Alafs*112 | II | 1 | II-3 | Germany | F | Yes (30) | −2.3 | NA | 24 y | −4.4 | Yes | NA | Yes | Convex facial profile, sloping forehead, marginally low-set and posteriorly rotated ears, small chin, and full lips, bilateral pes planus and hallux valgus | Farag, 2013 |
|  | c.1313G>A/p.Arg438His c.2864_2867delACAG/p.Asp955Alafs*112 | II | 1 | II-1 | Germany | F | NA | −2.3 | NA | 2 y | −3.3 | Yes | Yes | Yes | Sloping forehead | Farag, 2013 |
|  | c.1531G>A/p.Asp511Asn c.3469_3470delGC/p.Ala1157Cysfs*5 | NA27 | 1 | P 4 | NA | F | No | −2 | NA | 11 y | −3 | No | Yes | NA | NA | Ruaud, 2021 |
|  | c.1531G>A/p.Asp511Asn c.3469_3470delGC/p.Ala1157Cysfs*5 | NA28 | 1 | #33 | Belgium | F | NA | −2 | NA | 6 y | −4 | No | NA | Yes | NA | Duerinckx, 2021 |
|  | c.2486C>T/p.Thr829Ile c.1963dupT/p.Tyr655Leufs*18 | NA29 | 1 | P 2 | NA | M | NA | −1.5 | NA | 24 y 6 m | −3.5 | Yes | Yes | Yes | NA | Ruaud, 2021 |
|  | c.2584G>A/p.Gly862Ser c.2864_2867delACAG/p.Asp955Alafs*112 | NA30 | 1 | P 4 | Germany | NA | NA | NA | NA | NA | NA | NA | Yes | NA | NA | Poulton, 2014 |
| Frameshift  Frameshift | c.1711_1712insTA/p.Asn571Ilefs*27 c.2864_2867delACAG/p.Asp955Alafs*112 | c | 1 | case 3 | Poland | M | Yes (36) | −3.5 | No | 5 y | −6.5 | No | Yes | Yes | Narrow bitemporal diameter, coarse facial features, ptosis of the left eyelid | Slezak, 2021 |
|  | c.2083delA/p.Ser696Alafs*4 c.2746_2747delAG/p.Gln918Glyfs*18 | II | 1 | P II-1 | NA | M | Yes (30) | NA | Gestational diabetes | 8 y 10 m | −3 | Yes | Yes | Yes | NA | Murdock, 2011 |
|  | c.2083delA/p.Ser696Alafs*4 c.2746_2747delAG/p.Gln918Glyfs*18 | II | 1 | P II-2 | NA | M | No | NA | No | 4 y | −4 | No | Yes | NA | NA | Murdock, 2011 |
|  | c.2319delG/p.Ser774Valfs*19 c.3936dupC/p.Val1313Argfs*18 | F1 | 1 | case 1 | Turkey | F | NA | −2.5 | NA | 17 y | −7.7 | Yes | Yes | NA | No | Bolat, 2022 |
|  | c.2319delG/p.Ser774Valfs*19 c.3936dupC/p.Val1313Argfs*18 | F1 | 1 | case 2 | Turkey | F | NA | −3.2 | NA | 10 y | −5.1 | No | Yes | NA | Sloping forehead | Bolat, 2022 |
| **Variation types** | **Variations**  **(n = 93)** | **Family identification  (n = 100)** | **Number of patients  (n = 217)** | **Patient identification** | **Geographic origin (n = 21)** | **Sex** | **Microcephaly detected prior birth (term in WG), n = 19, available 19/217 (8.8%)** | **OFC at birth (SD), n = 54, available 54/217 (24.9%)** | **Pregnancy complications(n = 13)** | **Age at last examination** | **OFC at last examination (SD), n = 185, available 185/217 (85.2%)** | **Epilepsy, n = 81, positive available 81/217 (37.3%)** | **DD, n = 137, positive available 137/217 (63.1%)** | **ID/MR, n = 113, positive available 113/217 (52.1%)** | **Dysmorphysm, n = 77, available 77/217 (35.5%)** | **References(n = 44)** |
| Frameshift Nonsense | c.2963delC/p.Pro988Argfs*80 c.3163G>T/p.Glu1055* | NA31 | 1 | NA | China | F | Yes (?) | NA | NA | 1 y | NA | No | Yes | NA | NA | Yu, 2025 |
|  | c.3012_3034dup/p.Pro1012Glnfs*64 c.3124G>T/p.Gly1042* | NA32 | 1 | NA | NA | NA | NA | NA | NA | NA | NA | NA | NA | NA | NA | Stranneheim, 2021 |
|  | c.3620_3621delAG/p.Gln1207Argfs*29 c.1128C>A/p.Cys376* | NA33 | 1 | NA | China | F | NA | NA | NA | 14 y | NA | Yes | Yes | NA | Hypertelorism, Frontal inclination, macrotia, Left hypertropia | Ding, 2018 |
| Splicing site Frameshift | c.1642+2T>G/- c.1777_1778delGA/p.Asp593Hisfs*9 | a | 1 | case 1 | Poland | F | No | < −2 | Persistent foramen ovale | 4 m | NA | NA | Yes | NA | Prominent occiput, and upslanted palpebral fissures | Slezak, 2021 |
|  | c.1043+3A>G/- c.3383_3401del19/p.Ser1128Trpfs*164 | NA34 | 1 | #36 | Belgium | M | NA | −3.5 | NA | 28 y | −6 | Yes | NA | NA | Hearing  deficiency | Duerinckx, 2021 |
| Splicesite Nonsense | c.1550+3A>C/- c.2051C>G/p.Ser684* | NA35 | 1 | P287 | China | M | NA | NA | NA | 3 y 3 m | NA | NA | Yes | Yes | Abnormality of the skeletal system | Zhang, 2023 |
| Synonymous Nonsense | c.1521G>A/p.Leu507= c.2788C>T/p.Gln930* | NA36 | 2 | #37 | Belgium | F | NA | −2 | NA | 5 y 6 m | −3 | Yes | NA | Yes | NA | Duerinckx, 2021 |
|  | c.1521G>A/p.Leu507= c.2788C>T/p.Gln930* | NA37 | 1 | P 15 | NA | F | No | −1.5 | NA | 14 y 6 m | −3 | Yes | Yes | NA | NA | Ruaud, 2021 |
|  | c.1521G>A/p.Leu507= c.2788C>T/p.Gln930* | NA37 | 1 | P 16 | NA | F | No | −1.5 | NA | 4 y | −5 | Yes | Yes | NA | Strabismus | Ruaud, 2021 |
| Intron Missense | c.883-4_890del c.1684C>G/p.His562Asp | NA38 | 1 | NA | South Korea | F | Yes (?) | < −3 | Intrauterine growth restriction | 6 y | −6 | No | Yes | Yes | NA | Yi, 2019 |

Notes:

This table strictly preserves original authors' terminology.

The patient data were systematically categorized into two major groups based on Variation patterns: homozygous and heterozygous Variations.

Abbreviations: Yes: presence; No: absence, DD: developmental delay; F: female; ID: intellectual disability; MR: mental retardation; m: month; M: male; NA: Not available; SD: standard deviation; OFC: occipitofrontal circumference; WG : week of gestation; y: year.

Table S2 Radiographic imaging findings of *WDR62* gene variants in a total of 92 patients with MCPH2 reported in the literature and this study.

| **Variation type** | **Variations**  **(n = 74)** | **Family identification  (n = 69)** | **Patient  identification** | **Ventricles or extra cerebral spaces enlargement, n = 21**, proportion 21/92 (22.8%) | **corpus callosum abnormality, n = 33, proportion 33/92 (35.9%)** | **Gyral simplification, n = 31, proportion 31/92 (33.7%)** | **Pachygyria, n = 44, proportion 44/92 (47.8%)** | **Thickened Cortex, n = 26, proportion 26/92 (28.3%)** | **Neuronal heterotopia, n = 9, proportion 9/92 (9.8%)** | **Schizencephaly, n = 10 proportion 10/92 (10.9%)** | **Polymicrogyria, n = 23 proportion 23/92 (25.0%)** | **Lissencephaly, n =14 proportion 14/92 (15.2%)** | **Brainstem and cerebellum anomalies, n = 13 proportion 13/92 (14.1%)** | **Subcortical White matter anomalies, n =13 proportion 13/92 (14.1%)** | **Hippocampus abnormality, n = 8 proportion 8/92 (8.7%)** | **Other cortical malformations, n = 2 proportion 2/92 (2.2%)** | **References (n =33)** |
| --- | --- | --- | --- | --- | --- | --- | --- | --- | --- | --- | --- | --- | --- | --- | --- | --- | --- |
| **1. Homozygous** | | | | | | | | | | | | | | | | | |
| Missense | c.193G>A/p.Val65Met | PH-16900 | PH-16901 | NA | No | Yes | NA | NA | NA | NA | NA | NA | Yes | NA | NA | NA | Yu, 2010 |
|  | c.193G>A/p.Val65Met | PH-16900 | PH-16902 | NA | Yes | NA | NA | NA | NA | Yes | Yes | NA | NA | NA | NA | NA | Yu, 2010 |
|  | c.193G>A/p.Val65Met | PH-16900 | PH-16903 | NA | NA | NA | NA | NA | NA | Yes | Yes | NA | NA | NA | NA | NA | Yu, 2010 |
|  | c.359C>A/p.Ser120Tyr | NA1 | F43.1✝ | NA | NA | NA | Yes | NA | NA | Yes | NA | NA | NA | NA | NA | NA | Masih, 2022 |
|  | c.668T>C/p.Phe223Ser | NA2 | P1 | Yes | Yes | NA | Yes | Yes | NA | NA | NA | NA | Yes | Yes | NA | NA | Zombor, 2019 |
|  | c.668T>C/p.Phe223Ser | NA2 | P2 | Yes | No | No | Yes | Yes | Yes | NA | NA | NA | NA | Yes | No | NA | Zombor, 2019 |
|  | c.671G>C/p.Trp224Ser | NG 190 | NG 190-1 | Yes | Yes | NA | Yes | Yes | NA | Yes | Yes | NA | NA | NA | Yes | NA | Bilguvar, 2010 |
|  | c.671G>C/p.Trp224Ser | NG 190 | NG 190-5 | No | Yes | NA | Yes | Yes | NA | No | No | NA | NA | NA | Yes | NA | Bilguvar, 2010 |
|  | c.671G>C/p.Trp224Ser | NG 190 | NG 190-6 | No | Yes | NA | Yes | Yes | NA | No | No | NA | NA | NA | Yes | NA | Bilguvar, 2010 |
|  | c.883G>T/p.Val295Phe | NA3 | P 11 | No | No | No | Yes | No | No | No | No | No | No | Yes | No | No | Ruaud, 2021 |
|  | c.883G>T/p.Val295Phe | NA3 | P 12 | No | No | Yes | No | No | No | No | No | No | No | No | No | No | Ruaud, 2021 |
|  | c.1198G>A/p.Glu400Lys | IV | IV-8 | NA | NA | Yes | No | No | No | No | No | NA | NA | NA | NA | NA | Bacino, 2012 |
|  | c.1480G>A/p.Gly494Arg | NA4 | P 7 | No | No | No | No | No | No | No | No | No | No | Yes | No | No | Ruaud, 2021 |
|  | c.1526C>T/p.Ser509Leu | NA6 | P 17 | NA | NA | Yes | Yes | NA | NA | NA | NA | NA | NA | NA | NA | NA | Ruaud, 2021 |
|  | c.1576G>A/p.Glu526Lys | NG 537 | NG 537-1 | NA | Yes | NA | Yes | Yes | NA | No | No | NA | No | No | No | NA | Bilguvar, 2010 |
| **Variation type** | **Variations**  **(n = 74)** | **Family identification  (n = 69)** | **Patient  identification** | **Ventricles or extra cerebral spaces enlargement, n = 21**, proportion 21/92 (22.8%) | **corpus callosum abnormality, n = 33, proportion 33/92 (35.9%)** | **Gyral simplification, n = 31, proportion 31/92 (33.7%)** | **Pachygyria, n = 44, proportion 44/92 (47.8%)** | **Thickened Cortex, n = 26, proportion 26/92 (28.3%)** | **Neuronal heterotopia, n = 9, proportion 9/92 (9.8%)** | **Schizencephaly, n = 10 proportion 10/92 (10.9%)** | **Polymicrogyria, n = 23 proportion 23/92 (25.0%)** | **Lissencephaly, n =14 proportion 14/92 (15.2%)** | **Brainstem and cerebellum anomalies, n = 13 proportion 13/92 (14.1%)** | **Subcortical White matter anomalies, n =13 proportion 13/92 (14.1%)** | **Hippocampus abnormality, n = 8 proportion 8/92 (8.7%)** | **Other cortical malformations, n = 2 proportion 2/92 (2.2%)** | **References (n =33)** |
| Missense | c.1598A>G/ p.His533Arg | NA7 | III.2 | Yes | NA | NA | Yes | NA | Yes | NA | Yes | Yes | NA | No | NA | NA | Hajar, 2022 |
|  | c.1598A>G/ p.His533Arg | NA7 | III.1 | Yes | NA | NA | Yes | NA | Yes | NA | Yes | Yes | NA | Yes | Yes | NA | Hajar, 2022 |
|  | c.2030T>C/p.Leu677Pro | NA8 | P 8 | NA | Yes | NA | NA | NA | NA | Yes | Yes | Yes | NA | NA | NA | NA | Ruaud, 2021 |
|  | c.2588G>A/p.Arg863His | NA9 | P 3 | Yes | NA | NA | NA | NA | NA | NA | Yes | NA | NA | NA | NA | NA | Poulton, 2014 |
|  | c.2588G>A/p.Arg863His | NA10 | P 13 | No | No | No | Yes | NA | NA | NA | Yes | NA | Yes | NA | NA | NA | Ruaud, 2021 |
|  | c.2588G>A/p.Arg863His | NA10 | P 14 | No | No | No | Yes | Yes | No | No | Yes | NA | Yes | NA | NA | NA | Ruaud, 2021 |
|  | c.3878C>A/p.Ala1293Asp | IV | IV-1 | NA | NA | NA | NA | NA | NA | NA | NA | NA | Yes | NA | NA | NA | Naseer, 2017 |
| Nonsense | c.321dupT/p.Asn108* | 26 | A33021 | NA | Yes | NA | NA | NA | NA | NA | NA | NA | NA | NA | Yes | NA | Wang, 2023 |
|  | c.900C>A/p.Cys300* | IIS-43 | IV-1 | NA | NA | NA | Yes | No | Yes | NA | Yes | Yes | NA | NA | NA | NA | Bhat, 2011 |
|  | c.900C>A/p.Cys300* | IIS-43 | IV-2 | NA | NA | NA | Yes | No | NA | NA | Yes | NA | NA | NA | NA | NA | Bhat, 2011 |
|  | c.1319G>A/p.Trp440* | 9506900 | 202 | Yes | NA | NA | NA | NA | NA | NA | NA | NA | Yes | NA | NA | NA | Abolhassani, 2024 |
|  | c.1408C>T/p.Gln470* | NG 294 | NG 294-1 | NA | Yes | NA | Yes | Yes | NA | No | No | NA | NA | NA | Yes | NA | Bilguvar, 2010 |
|  | c.1576G>T/p.Glu526* | NG 30 | NG 30-1 | NA | Yes | NA | Yes | Yes | NA | No | No | NA | NA | NA | Yes | NA | Bilguvar, 2010 |
|  | c.1605dupT/p.Glu536* | NA12 | P 1 | Yes | Yes | Yes | Yes | Yes | NA | NA | NA | Yes | NA | NA | No | NA | Poulton, 2014 |
|  | c.1605dupT/p.Glu536* | F2 | Case 3 | NA | Yes | Yes | NA | NA | NA | NA | NA | NA | NA | NA | NA | NA | Bolat, 2022 |
|  | c.1605dupT/p.Glu536* | F3 | case 5 | NA | NA | Yes | NA | NA | NA | Yes | Yes | NA | NA | NA | NA | NA | Bolat, 2022 |
| **Variation type** | **Variations**  **(n = 74)** | **Family identification  (n = 69)** | **Patient  identification** | **Ventricles or extra cerebral spaces enlargement, n = 21**, proportion 21/92 (22.8%) | **corpus callosum abnormality, n = 33, proportion 33/92 (35.9%)** | **Gyral simplification, n = 31, proportion 31/92 (33.7%)** | **Pachygyria, n = 44, proportion 44/92 (47.8%)** | **Thickened Cortex, n = 26, proportion 26/92 (28.3%)** | **Neuronal heterotopia, n = 9, proportion 9/92 (9.8%)** | **Schizencephaly, n = 10 proportion 10/92 (10.9%)** | **Polymicrogyria, n = 23 proportion 23/92 (25.0%)** | **Lissencephaly, n =14 proportion 14/92 (15.2%)** | **Brainstem and cerebellum anomalies, n = 13 proportion 13/92 (14.1%)** | **Subcortical White matter anomalies, n =13 proportion 13/92 (14.1%)** | **Hippocampus abnormality, n = 8 proportion 8/92 (8.7%)** | **Other cortical malformations, n = 2 proportion 2/92 (2.2%)** | **References (n =33)** |
| Nonsense | c.1605dupT/p.Glu536* | F4 | case 6 | NA | NA | NA | Yes | NA | NA | NA | NA | Yes | NA | NA | NA | NA | Bolat, 2022 |
|  | c.1605dupT/p.Glu536* | NA13 | P 6 | NA | Yes | NA | Yes | No | NA | NA | NA | NA | NA | NA | NA | NA | Poulton, 2014 |
|  | c.1942C>T/p.Gln648* | MCP67 | IV-5 | Yes | NA | Yes | No | Yes | NA | NA | NA | NA | NA | NA | NA | NA | Kousar, 2011 |
|  | c.2956C>T/p.Gln986* | F5 | 7 | NA | NA | NA | NA | NA | NA | NA | NA | Yes | No | NA | NA | NA | Bolat, 2022 |
|  | c.3790G>T/p.Glu1264* | NA14 | P 9 | No | Yes | Yes | Yes | No | No | No | No | No | No | No | No | No | Ruaud, 2021 |
|  | c.4345C>T/p.Gln1449* | NA15 | P 5 | No | NA | Yes | Yes | No | No | No | No | No | No | No | No | No | Ruaud, 2021 |
| Synonymous | c.390G>A/p.Glu130= | IV | IV-4 | Yes | No | No | No | No | No | No | No | Yes | No | No | No | No | Bastaki, 2016 |
|  | c.390G>A/p.Glu130= | IV | IV-5 | No | No | No | Yes | No | No | No | No | Yes | No | No | No | No | Bastaki, 2016 |
|  | c.2115C>G/p.Gly705= | M196 | IV:1 | Yes | NA | NA | NA | NA | NA | NA | NA | NA | Yes | NA | NA | NA | Hu, 2014 |
|  | c.2115C>G/p.Gly705= | M196 | IV:4 | Yes | NA | NA | NA | NA | NA | NA | NA | NA | Yes | NA | NA | NA | Hu, 2014 |
| Frameshift | c.363delT/p.Asp122Metfs*5 | LIS-900 | LIS-901 | NA | NA | Yes | NA | NA | NA | NA | NA | NA | NA | NA | NA | NA | Yu, 2010 |
|  | c.363delT/p.Asp122Metfs*5 | LIS-900 | LIS-902 | Yes | NA | Yes | NA | NA | NA | NA | NA | NA | NA | NA | NA | NA | Yu, 2010 |
|  | c.363delT/p.Asp122Metfs*5 | LIS-900 | LIS-903 | No | Yes | Yes | NA | Yes | Yes | NA | NA | NA | NA | NA | NA | NA | Yu, 2010 |
|  | c.384_385delAG/p.Asn131Trpfs*3 | F6 | 8 | NA | NA | NA | Yes | Yes | NA | Yes | NA | NA | NA | NA | NA | NA | Bolat, 2022 |
|  | c.535dup/p.Met179Asnfs*22 | IIS-36 | IV-2 | NA | NA | NA | Yes | Yes | NA | NA | NA | NA | NA | NA | NA | NA | Bhat, 2011 |
| **Variation type** | **Variations**  **(n = 74)** | **Family identification  (n = 69)** | **Patient  identification** | **Ventricles or extra cerebral spaces enlargement, n = 21**, proportion 21/92 (22.8%) | **corpus callosum abnormality, n = 33, proportion 33/92 (35.9%)** | **Gyral simplification, n = 31, proportion 31/92 (33.7%)** | **Pachygyria, n = 44, proportion 44/92 (47.8%)** | **Thickened Cortex, n = 26, proportion 26/92 (28.3%)** | **Neuronal heterotopia, n = 9, proportion 9/92 (9.8%)** | **Schizencephaly, n = 10 proportion 10/92 (10.9%)** | **Polymicrogyria, n = 23 proportion 23/92 (25.0%)** | **Lissencephaly, n =14 proportion 14/92 (15.2%)** | **Brainstem and cerebellum anomalies, n = 13 proportion 13/92 (14.1%)** | **Subcortical White matter anomalies, n =13 proportion 13/92 (14.1%)** | **Hippocampus abnormality, n = 8 proportion 8/92 (8.7%)** | **Other cortical malformations, n = 2 proportion 2/92 (2.2%)** | **References (n =33)** |
| Frameshift | c.1143delC/p.His381Glnfs*49 | V | V-3 | Yes | Yes | NA | NA | NA | NA | NA | NA | NA | Yes | NA | NA | NA | Memon, 2013 |
|  | c.1973_1974del/p.Val658Glufs*14 | 13 | IV-3 | NA | NA | NA | No | Yes | NA | NA | Yes | NA | NA | NA | NA | NA | Kvarnung, 2018 |
|  | c.2527dupG/p.Asp843Glyfs*3 | MCP1 | MCP1-6 | Yes | NA | NA | No | Yes | NA | NA | NA | NA | NA | NA | NA | NA | Rupp, 2014 |
|  | c.2864_2867delACAG/p.Asp955Alafs*112 | NG1406 | NG1406-1 | NA | Yes | NA | Yes | Yes | NA | NA | NA | NA | NA | NA | NA | NA | Sgourdou, 2017 |
|  | c.2864_2867delACAG/p.Asp955Alafs*112 | NA16 | P 2 | NA | Yes | Yes | No | Yes | NA | NA | NA | NA | No | NA | NA | NA | Poulton, 2014 |
|  | c.3839_3855delGCCAAGAGCCTGCCCTG/p.Gly1280Alafs*21 | MC-1600 | MC-1601 | No | Yes | NA | NA | NA | NA | NA | NA | NA | Yes | NA | NA | NA | Yu, 2010 |
|  | c.3839_3855delGCCAAGAGCCTGCCCTG/p.Gly1280Alafs*21 | NG 339 | NG 339-1 | NA | Yes | NA | Yes | Yes | NA | No | No | NA | NA | NA | Yes | NA | Bilguvar, 2010 |
|  | c.3936dupC/p.Val1313Argfs*18 | PC12 | NA | NA | NA | Yes | NA | Yes | NA | NA | NA | NA | NA | NA | NA | NA | Nicholas, 2010 |
|  | c.3936dupC/p.Val1313Argfs*18 | LIS-2600 | LIS-2601 | NA | NA | Yes | NA | NA | NA | NA | NA | NA | NA | NA | NA | NA | Yu, 2010 |
|  | c.3936dupC/p.Val1313Argfs*18 | LIS-2600 | LIS-2602 | NA | Yes | Yes | NA | NA | NA | NA | Yes | NA | NA | NA | NA | NA | Yu, 2010 |
|  | c.3936dupC/p.Val1313Argfs*18 | NA17 | #38 | NA | NA | Yes | Yes | No | NA | NA | NA | Yes | NA | NA | NA | No | Duerinckx, 2021 |
|  | c.3936dupC/p.Val1313Argfs*18 | NA18 | P 3 | No | No | Yes | Yes | No | No | No | No | No | No | No | No | No | Ruaud, 2021 |
|  | c.4205_4208delTGCC/p.Val1402Glyfs*12 | NG 26 | NG 26-1 | NA | Yes | NA | Yes | Yes | NA | No | Yes | NA | NA | NA | No | NA | Bilguvar, 2010 |
|  | c.4205_4208delTGCC/p.Val1402Glyfs*12 | NG 891 | NG 891-1 | NA | Yes | NA | Yes | Yes | NA | No | No | NA | NA | NA | No | NA | Bilguvar, 2010 |
| **Variation type** | **Variations**  **(n = 74)** | **Family identification  (n = 69)** | **Patient  identification** | **Ventricles or extra cerebral spaces enlargement, n = 21**, proportion 21/92 (22.8%) | **corpus callosum abnormality, n = 33, proportion 33/92 (35.9%)** | **Gyral simplification, n = 31, proportion 31/92 (33.7%)** | **Pachygyria, n = 44, proportion 44/92 (47.8%)** | **Thickened Cortex, n = 26, proportion 26/92 (28.3%)** | **Neuronal heterotopia, n = 9, proportion 9/92 (9.8%)** | **Schizencephaly, n = 10 proportion 10/92 (10.9%)** | **Polymicrogyria, n = 23 proportion 23/92 (25.0%)** | **Lissencephaly, n =14 proportion 14/92 (15.2%)** | **Brainstem and cerebellum anomalies, n = 13 proportion 13/92 (14.1%)** | **Subcortical White matter anomalies, n =13 proportion 13/92 (14.1%)** | **Hippocampus abnormality, n = 8 proportion 8/92 (8.7%)** | **Other cortical malformations, n = 2 proportion 2/92 (2.2%)** | **References (n =33)** |
| Splicesite | c.1043+1G>A/- | LIS-2500 | LIS-2501 | NA | Yes | Yes | NA | NA | Yes | NA | NA | NA | NA | NA | NA | NA | Yu, 2010 |
|  | c.2739+2T>C/- | NA20 | P 1 | No | Yes | Yes | Yes | No | No | No | No | No | No | No | No | No | Ruaud, 2021 |
|  | c.3335+1G>C/- | NA21 | NA | NA | NA | NA | NA | NA | NA | NA | Yes | NA | NA | NA | NA | Graynowhite matter blurring | Nardello, 2018 |
|  | c.1372-1delG/- | Family A | VI-9 | Yes | No | Yes | Yes | NA | NA | NA | NA | Yes | NA | NA | NA | NA | Aslam, 2024 |
| Intron | c.2867+4_c.2867+7delGGGTC | MC-1400 | MC-1403 | NA | Yes | NA | NA | NA | Yes | NA | NA | NA | No | NA | NA | NA | Yu, 2010 |
| **2. compound heterozygous** | | | | | | | | | | | | | | | | | |
| Missense  Missense | c.28G>T/p.Ala10Ser c.189G>T/p.Glu63Asp | NA22 | NA | Yes | Yes | NA | NA | NA | NA | Yes | NA | NA | Yes | NA | Yes | Temporal lobe dysplasia, leukodystrophy | Banerjee, 2016 |
|  | c.797C>T/p.Ala266Val c.1102G>A/p.Asp368Asn | IV | IV-1 | Yes | NA | NA | NA | NA | NA | NA | NA | NA | NA | Yes | NA | NA | Naseer, 2019 |
|  | c.797C>T/p.Ala266Val c.1102G>A/p.Asp368Asn | IV | IV-2 | NA | NA | NA | NA | NA | NA | NA | NA | NA | NA | Yes | NA | NA | Naseer, 2019 |
|  | c.836G>A/p.Cys279Tyr c.1480G>A/p.Gly494Arg | 25 | CS19 | NA | NA | NA | NA | NA | NA | NA | Yes | NA | NA | Yes | NA | NA | Stutterd, 2021 |
|  | c.836G>A/p.Cys279Tyr c.1480G>A/p.Gly494Arg | 26 | CS20 | NA | NA | NA | NA | NA | NA | NA | Yes | NA | NA | Yes | NA | NA | Stutterd, 2021 |
|  | c.4162G>A/p.Gly1388Ser c.4178G>T/p.Ser1393Ile | NA24 | P 6 | No | No | No | No | No | No | No | No | No | No | Yes | No | No | Ruaud, 2021 |
| **Variation type** | **Variations**  **(n = 74)** | **Family identification  (n = 69)** | **Patient  identification** | **Ventricles or extra cerebral spaces enlargement, n = 21**, proportion 21/92 (22.8%) | **corpus callosum abnormality, n = 33, proportion 33/92 (35.9%)** | **Gyral simplification, n = 31, proportion 31/92 (33.7%)** | **Pachygyria, n = 44, proportion 44/92 (47.8%)** | **Thickened Cortex, n = 26, proportion 26/92 (28.3%)** | **Neuronal heterotopia, n = 9, proportion 9/92 (9.8%)** | **Schizencephaly, n = 10 proportion 10/92 (10.9%)** | **Polymicrogyria, n = 23 proportion 23/92 (25.0%)** | **Lissencephaly, n =14 proportion 14/92 (15.2%)** | **Brainstem and cerebellum anomalies, n = 13 proportion 13/92 (14.1%)** | **Subcortical White matter anomalies, n =13 proportion 13/92 (14.1%)** | **Hippocampus abnormality, n = 8 proportion 8/92 (8.7%)** | **Other cortical malformations, n = 2 proportion 2/92 (2.2%)** | **References (n =33)** |
| Missense  Nonsense | c.194T>A/p.Val65Glu c.4273C>T/p.Gln1425* | b | Case 2 | No | Yes | Yes | No | No | No | No | No | No | No | No | No | NA | Slezak, 2021 |
|  | c.643A>C/p.Thr215Pro c.1128C>A/p.Cys376* | NA25 | Ⅱ:2 | No | Yes | No | Yes | Yes | No | Yes | No | No | No | No | No | No | This stdy |
|  | c.643A>C/p.Thr215Pro c.1128C>A/p.Cys376* | NA25 | Ⅱ:3 | No | Yes | No | Yes | Yes | No | No | No | Yes | No | No | No | No | This stdy |
|  | c.1531G>A/p.Asp511Asn c.4345C>T/p.Gln1449* | NA26 | #32 | NA | NA | NA | Yes | NA | NA | NA | NA | Yes | NA | NA | NA | NA | Duerinckx, 2021 |
| Missense Frameshift | c.1313G>A/p.Arg438His c.2864_2867delACAG/p.Asp955Alafs*112 | II | II-1 | Yes | No | No | No | No | No | No | No | NA | NA | NA | NA | NA | Farag, 2013 |
|  | c.1313G>A/p.Arg438His c.2864_2867delACAG/p.Asp955Alafs*112 | II | II-3 | NA | Yes | Yes | No | No | No | No | No | NA | Yes | NA | NA | NA | Farag, 2013 |
|  | c.1531G>A/p.Asp511Asn c.3469_3470delGC/p.Ala1157Cysfs*5 | NA27 | P 4 | NA | NA | NA | Yes | No | NA | NA | NA | NA | NA | Yes | NA | NA | Ruaud, 2021 |
|  | c.1531G>A/p.Asp511Asn c.3469_3470delGC/p.Ala1157Cysfs*5 | NA28 | #33 | NA | NA | NA | Yes | NA | NA | NA | NA | Yes | NA | NA | NA | NA | Duerinckx, 2021 |
|  | c.2486C>T/p.Thr829Ile c.1963dupT/p.Tyr655Leufs*18 | NA29 | P 2 | NA | NA | NA | Yes | No | Yes | NA | NA | NA | NA | NA | NA | NA | Ruaud, 2021 |
|  | c.2584G>A/p.Gly862Ser c.2864_2867delACAG/p.Asp955Alafs*112 | NA30 | P 4 | NA | NA | NA | Yes | NA | NA | NA | Yes | NA | NA | NA | NA | NA | Poulton, 2014 |
| Frameshift Frameshift | c.1711_1712insTA/p.Asn571Ilefs*27 c.2864_2867delACAG/p.Asp955Alafs*112 | c | case 3 | Yes | No | Yes | Yes | Yes | NA | NA | Yes | NA | NA | NA | NA | NA | Slezak, 2021 |
|  | c.2083delA/p.Ser696Alafs*4 c.2746_2747delAG/p.Gln918Glyfs*18 | II | P II-1 | NA | NA | Yes | No | No | No | No | Yes | NA | NA | NA | NA | NA | Murdock, 2011 |
|  | c.2083delA/p.Ser696Alafs*4 c.2746_2747delAG/p.Gln918Glyfs*18 | II | P II-2 | NA | NA | No | No | No | Yes | No | Yes | NA | NA | NA | NA | NA | Murdock, 2011 |
| **Variation type** | **Variations**  **(n = 74)** | **Family identification  (n = 69)** | **Patient  identification** | **Ventricles or extra cerebral spaces enlargement, n = 21**, proportion 21/92 (22.8%) | **corpus callosum abnormality, n = 33, proportion 33/92 (35.9%)** | **Gyral simplification, n = 31, proportion 31/92 (33.7%)** | **Pachygyria, n = 44, proportion 44/92 (47.8%)** | **Thickened Cortex, n = 26, proportion 26/92 (28.3%)** | **Neuronal heterotopia, n = 9, proportion 9/92 (9.8%)** | **Schizencephaly, n = 10 proportion 10/92 (10.9%)** | **Polymicrogyria, n = 23 proportion 23/92 (25.0%)** | **Lissencephaly, n =14 proportion 14/92 (15.2%)** | **Brainstem and cerebellum anomalies, n = 13 proportion 13/92 (14.1%)** | **Subcortical White matter anomalies, n =13 proportion 13/92 (14.1%)** | **Hippocampus abnormality, n = 8 proportion 8/92 (8.7%)** | **Other cortical malformations, n = 2 proportion 2/92 (2.2%)** | **References (n =33)** |
| Frameshift Nonsense | c.2963delC/p.Pro988Argfs*80 c.3163G>T/p.Glu1055* | NA31 | NA | NA | NA | NA | Yes | NA | NA | NA | NA | NA | NA | NA | NA | NA | Yu, 2025 |
|  | c.3620_3621delAG/p.Gln1207Argfs*29 c.1128C>A/p.Cys376* | NA33 | NA | Yes | Yes | NA | Yes | Yes | No | No | No | No | No | No | No | NA | Ding, 2018 |
| Splicing site Frameshift | c.1642+2T>G/- c.1777_1778delGA/p.Asp593Hisfs*9 | a | case 1 | No | Yes | Yes | No | No | No | Yes | Yes | No | No | No | No | No | Slezak, 2021 |
|  | c.1550+3A>C/- c.2051C>G/p.Ser684* | NA34 | P 287 | No | No | No | Yes | No | No | No | No | No | No | No | No | No | Zhang, 2023 |
| Synonymous Nonsense | c.1521G>A/p.Leu507= c.2788C>T/p.Gln930* | NA36 | #37 | NA | NA | Yes | NA | NA | NA | NA | NA | NA | NA | NA | No | NA | Duerinckx, 2021 |
|  | c.1521G>A/p.Leu507= c.2788C>T/p.Gln930* | NA37 | P 15 | NA | NA | Yes | NA | NA | NA | NA | NA | NA | NA | Yes | NA | NA | Ruaud, 2021 |
|  | c.1521G>A/p.Leu507= c.2788C>T/p.Gln930* | NA37 | P 16 | NA | NA | Yes | NA | NA | NA | NA | NA | NA | NA | Yes | NA | NA | Ruaud, 2021 |
| Intron Missense | c.883-4_890del c.1684C>G/p.His562Asp | NA38 | NA | NA | NA | Yes | No | No | No | No | No | NA | NA | NA | NA | NA | Yi, 2019 |

Notes:

This table strictly preserves original authors' terminology.

The patient data were systematically categorized into two major groups based on Variations patterns: homozygous and heterozygous Variations.

Abbreviations: Yes: presence; No: absence, NA: Not available.

*References*:

1. Yu TW, Mochida GH, Tischfield DJ, et al. Mutations in *WDR62*, encoding a centrosome-associated protein, cause microcephaly with simplified gyri and abnormal cortical architecture. *Nat Genet* 2010; 42:1015-20.

2. Nicholas AK, Khurshid M, Désir J, et al. *WDR62* is associated with the spindle pole and is mutated in human microcephaly. *Nat Genet* 2010; 42:1010-4.

3. Sajid Hussain M, Marriam Bakhtiar S, Farooq M, et al. Genetic heterogeneity in Pakistani microcephaly families. *Clin Genet* 2013; 83:446-51.

4. Ahmad I, Baig SM, Abdulkareem AR, et al. Genetic heterogeneity in Pakistani microcephaly families revisited. *Clin Genet* 2017; 92:62-68.

5. Masih S, Moirangthem A, Shambhavi A, et al. Deciphering the molecular landscape of microcephaly in 87 Indian families by exome sequencing. *Eur J Med Genet* 2022; 65:104520.

6. Zombor M, Kalmár T, Nagy N, et al. A novel *WDR62* missense mutation in microcephaly with abnormal cortical architecture and review of the literature. *J Appl Genet* 2019; 60:151-62.

7. Bilgüvar K, Oztürk AK, Louvi A, et al. Whole-exome sequencing identifies recessive *WDR62* mutations in severe brain malformations. *Nature* 2010; 467:207-10.

8. Ruaud L, Drunat S, Elmaleh-Bergès M, et al. Neurological outcome in *WDR62* primary microcephaly. *Dev Med Child Neurol* 2022; 64:509-17.

9. Bacino CA, Arriola LA, Wiszniewska J, et al. *WDR62* missense mutation in a consanguineous family with primary microcephaly. *Am J Med Genet A* 2012; 158a:622-5.

10. Kousar R, Hassan MJ, Khan B, et al. Mutations in *WDR62* gene in Pakistani families with autosomal recessive primary microcephaly. *BMC Neurol* 2011; 11:119.

11. Duerinckx S, Désir J, Perazzolo C, et al. Phenotypes and genotypes in non-consanguineous and consanguineous primary microcephaly: High incidence of epilepsy. *Mol Genet Genomic Med* 2021; 9:e1768.

12. Aryan H, Zokaei S, Farhud D, et al. Novel phenotype and genotype spectrum of *WDR62* in two patients with associated primary autosomal recessive microcephaly. *Ir J Med Sci* 2022; 191:2733-41.

13. Rasool S, Baig JM, Moawia A, et al. An update of pathogenic variants in *ASPM*, *WDR62*, *CDK5RAP2*, *STIL*, *CENP*J, and *CEP135* underlying autosomal recessive primary microcephaly in 32 consanguineous families from Pakistan. *Mol Genet Genomic Med* 2020; 8:e1408.

14. Poulton CJ, Schot R, Seufert K, et al. Severe presentation of *WDR62* mutation: is there a role for modifying genetic factors? *Am J Med Genet A* 2014; 164a:2161-71.

15. Naseer MI, Rasool M, Sogaty S, et al. A novel *WDR62* mutation causes primary microcephaly in a large consanguineous Saudi family. *Ann Saudi Med* 2017; 37:148-53.

16. Wang Y, Fu F, Lei T, et al. Genetic diagnosis of fetal microcephaly at a single tertiary center in China. *Front Genet* 2023; 14:1112153.

17. Bhat V, Girimaji SC, Mohan G, et al. Mutations in *WDR62*, encoding a centrosomal and nuclear protein, in Indian primary microcephaly families with cortical malformations. *Clin Genet* 2011; 80:532-40.

18. Cherkaoui Jaouad I, Zrhidri A, Jdioui W, et al. A novel non sense mutation in *WDR62* causes autosomal recessive primary microcephaly: a case report. *BMC Med Genet* 2018; 19:118.

19. Abolhassani A, Fattahi Z, Beheshtian M, et al. Clinical application of next generation sequencing for Mendelian disease diagnosis in the Iranian population. *NPJ Genom Med* 2024; 9:12.

20. Bolat H, Sağer SG, Türkyılmaz A, et al. Autosomal Recessive Primary Microcephaly (MCPH) and Novel Pathogenic Variants in *ASPM* and *WDR62* Genes. *Mol Syndromol* 2022; 13:363-69.

21. Wang R, Khan A, Han S, et al. Molecular analysis of 23 Pakistani families with autosomal recessive primary microcephaly using targeted next-generation sequencing. *J Hum Genet* 2017; 62:299-304.

22. McSherry M, Masih KE, Elcioglu NH, et al. Identification of candidate gene *FAM183A* and novel pathogenic variants in known genes: High genetic heterogeneity for autosomal recessive intellectual disability. *PLoS One* 2018; 13:e0208324.

23. Bastaki F, Mohamed M, Nair P, et al. Novel splice-site mutation in *WDR62* revealed by whole-exome sequencing in a Sudanese family with primary microcephaly. *Congenit Anom (Kyoto)* 2016; 56:135-7.

24. Hu H, Wienker TF, Musante L, et al. Integrated sequence analysis pipeline provides one-stop solution for identifying disease-causing mutations. *Hum Mutat* 2014; 35:1427-35.

25. Memon MM, Raza SI, Basit S, et al. A novel *WDR62* mutation causes primary microcephaly in a Pakistani family. *Mol Biol Rep* 2013; 40:591-5.

26. McDonell LM, Warman Chardon J, Schwartzentruber J, et al. The utility of exome sequencing for genetic diagnosis in a familial microcephaly epilepsy syndrome. *BMC Neurol* 2014; 14:22.

27. Kvarnung M, Taylan F, Nilsson D, et al. Genomic screening in rare disorders: New mutations and phenotypes, highlighting ALG14 as a novel cause of severe intellectual disability. *Clin Genet* 2018; 94:528-37.

28. Rupp V, Rauf S, Naveed I, et al. A novel single base pair duplication in *WDR62* causes primary microcephaly. *BMC Med Genet* 2014; 15:107.

29. Sgourdou P, Mishra-Gorur K, Saotome I, et al. Disruptions in asymmetric centrosome inheritance and *WDR62*-Aurora kinase B interactions in primary microcephaly. *Sci Rep* 2017; 7:43708.

30. Aslam K, Saeed A, Saeed HI, et al. Molecular genetics, neuroimaging outcomes, and structural analyses of novel and recurrent variants of *WDR62* gene in two consanguineous Pakistani families with autosomal recessive primary microcephaly. *Mol Biol Rep* 2024; 51:783.

31. Nardello R, Fontana A, Antona V, et al. A novel mutation of *WDR62* gene associated with severe phenotype including infantile spasm, microcephaly, and intellectual disability. *Brain Dev* 2018; 40:58-64.

32. Banerjee S, Chen H, Huang H, et al. Novel mutations c.28G>T (p.Ala10Ser) and c.189G>T (p.Glu63Asp) in *WDR62* associated with early onset acanthosis and hyperkeratosis in a patient with autosomal recessive microcephaly type 2. *Oncotarget* 2016; 7:78363-71.

33. Li N, Zhou P, Tang H, et al. In-depth analysis reveals complex molecular aetiology in a cohort of idiopathic cerebral palsy. *Brain* 2022; 145:119-41.

34. Naseer MI, Rasool M, Abdulkareem AA, et al. Novel compound heterozygous mutations in *WDR62* gene leading to developmental delay and Primary Microcephaly in Saudi Family. *Pak J Med Sci* 2019; 35:764-70.

35. Stutterd CA, Brock S, Stouffs K, et al. Genetic heterogeneity of polymicrogyria: study of 123 patients using deep sequencing. *Brain Commun* 2021; 3:fcaa221.

36. Slezak R, Smigiel R, Obersztyn E, et al. Further Delineation of Phenotype and Genotype of Primary Microcephaly Syndrome with Cortical Malformations Associated with Mutations in the *WDR62* Gene. *Genes (Basel)* 2021; 12:

37. Miyamoto T, Akutsu SN, Fukumitsu A, et al. PLK1-mediated phosphorylation of *WDR62*/MCPH2 ensures proper mitotic spindle orientation. *Hum Mol Genet* 2017; 26:4429-40.

38. Farag HG, Froehler S, Oexle K, et al. Abnormal centrosome and spindle morphology in a patient with autosomal recessive primary microcephaly type 2 due to compound heterozygous *WDR62* gene mutation. *Orphanet J Rare Dis* 2013; 8:178.

39. Murdock DR, Clark GD, Bainbridge MN, et al. Whole-exome sequencing identifies compound heterozygous mutations in *WDR62* in siblings with recurrent polymicrogyria. *Am J Med Genet A* 2011; 155a:2071-7.

40. Yu L, Wang X, Liu L, et al. A case of primary microcephaly associated with compound heterozygous variants of *WDR62* gene. *Zhonghua Yi Xue Yi Chuan Xue Za Zhi* 2025; 42:175-79.

41. Stranneheim H, Lagerstedt-Robinson K, Magnusson M, et al. Integration of whole genome sequencing into a healthcare setting: high diagnostic rates across multiple clinical entities in 3219 rare disease patients. *Genome Med* 2021; 13:40.

42. Ding H-K, Zhang Y, Zeng Y-K, et al. The second reported case in China of microcephaly associated with compound heterozygous mutations in the *WDR62* Gene. *Chinese Journal of Prenatal Diagnosis (Electronic Edition)* 2018; 10:53-56.

43. Zhang H, Chen X, Tan H, et al. The exploration of genetic aetiology and diagnostic strategy for 321 Chinese individuals with intellectual disability. *Clin Chim Acta* 2023; 538:94-103.

44. Yi YG, Lee DW, Kim J, et al. Two Novel Mutations (c.883-4_890del and c.1684C>G) of *WDR62* Gene Associated With Autosomal Recessive Primary Microcephaly: A Case Report. *Front Pediatr* 2019; 7:457.
